# Multivariate Approaches in Quantitative Structure–Property Relationships Study for the Photostability Assessment of 1,4-Dihydropyridine Derivatives

**DOI:** 10.3390/pharmaceutics16020206

**Published:** 2024-01-31

**Authors:** Martina Chieffallo, Michele De Luca, Fedora Grande, Maria Antonietta Occhiuzzi, Miyase Gözde Gündüz, Antonio Garofalo, Giuseppina Ioele

**Affiliations:** 1 Department of Pharmacy, Health and Nutritional Sciences, University of Calabria, 87036 Rende, Italy; martina.chieffallo@unical.it (M.C.); michele.deluca@unical.it (M.D.L.); fedora.grande@unical.it (F.G.); mariaantonietta.occhiuzzi@unical.it (M.A.O.); antonio.garofalo@unical.it (A.G.); 2 Department of Pharmaceutical Chemistry, Faculty of Pharmacy, Hacettepe University, 06100 Ankara, Turkey; miyasegunduz@yahoo.com

**Keywords:** photodegradation study, QSPR study, multivariate curve resolution, ordinary least squares, molecular descriptors, ICH rules, calcium channel blockers

## Abstract

1,4-dihydropyridines (1,4-DHPs) are widely recognized as highly effective L-type calcium channel blockers with significant therapeutic benefits in the treatment of cardiovascular disorders. 1,4-DHPs can also target T-type calcium channels, making them promising drug candidates for neurological conditions. When exposed to light, all 1,4-DHPs tend to easily degrade, leading to an oxidation product derived from the aromatization of the dihydropyridine ring. Herein, the elaboration of a quantitative structure–property relationships (QSPR) model was carried out by correlating the light sensitivity of structurally different 1,4-DHPs with theoretical molecular descriptors. Photodegradation experiments were performed by exposing the drugs to a Xenon lamp following the ICH rules. The degradation was monitored by spectrophotometry, and experimental data were elaborated by Multivariate Curve Resolution (MCR) methodologies to assess the kinetic rates. The results were confirmed by the HPLC-DAD method. PaDEL-Descriptor software was used to calculate molecular descriptors and fingerprints related to the chemical structures. Seventeen of the 1875 molecular descriptors were selected and correlated to the photodegradation rate by means of the Ordinary Least Squares (OLS) algorithm. The chemometric model is useful to predict the photosensitivity of other 1,4-DHP derivatives with a very low relative error percentage of 5.03% and represents an effective tool to design new analogs characterized by higher photostability.

## 1. Introduction

Quantitative structure–property relationships (QSPR) are mathematical relationships that relate the chemical structure of a molecule to its physical or chemical properties [1]. The study of these relationships has always represented a crucial step in the pharmaceutical field, as it can often allow the avoidance of expensive biological testing or the carrying out of demanding experiments on a certain physico-chemical property.

Multivariate analytical methods can extract a great deal of information from a data set, and this approach becomes even more significant when the molecules being studied are dangerous materials or unstable compounds [2]. Chemometrics [3,4] is an efficient method to describe how a given physicochemical property varies as a function of the characteristics of the chemical structure of a molecule.

Molecular descriptors can be defined as mathematical representations of the properties of molecules that can be generated by algorithms [5]. In accordance with the definition of Todeschini and Consonni [6], it can distinguish experimental measures such as logP, molar refractivity, dipole moment, and theoretical molecular descriptors, which derive from a symbolic representation of the molecule. As an example, logP is a quantitative representation of the lipophilicity of the molecules, and it is obtained by measuring the partition of a compound between an aqueous and a lipophilic phase, which usually consists of water/n-octanol [5]. Theoretical molecular descriptors can be further classified according to the level of molecular representation required to calculate the descriptor. One-dimensional (1D) descriptors are the simplest type and stand for information calculated from the molecular formula of the molecule (e.g., molecular weight); two-dimensional (2D) descriptors usually represent molecular information regarding the size, shape, and electronic distribution of the molecule; and three-dimensional (3D) descriptors typically describe properties related to the 3D conformation of the molecule, such as intramolecular hydrogen bonding [7].

Accordingly, the calculated descriptors can be used to elaborate a QSPR multivariate model capable of predicting responses of interest for new compounds, such as the identification of specific parameters that influence a property of the molecules or the prediction of the behavior of other molecules belonging to the same series of compounds.

In the last decade, QSPR methods have been frequently applied to study photodegradation processes, especially in environmental risk assessment. Experimental studies along with QSAR/QSPR models have been used to determine the physiochemical properties of persistent organic pollutants responsible for serious environmental and health problems. The estimated properties have been extensively used to predict the environmental fate and transportation of chemicals [8]. As an example, using experiments and theoretical calculations, a QSPR model was developed to predict the kinetics and reaction mechanism of the photolysis of 25 individual polychlorinated diphenyl sulfides. In this case, the photodegradation process was significantly influenced by the dipole moment and the ELUMO–EHOMO descriptors [9]. Recently, Villaverde et al. developed QSAR/QSPR models to predict the presence of transformation products of an herbicide, alloxydim, during drinking water treatment processes such as chlorination or chloramination [10].

In the pharmaceutical field, the use of QSPR elaborations to describe the photodegradation process of drugs is more recent, and only a few applications are reported in the literature. Buglak et al. have developed a QSPR approach to predict the efficiency of singlet oxygen generation by free-base porphyrins and metalloporphyrins. These compounds are used as photosensitizers in photocatalysis and photodynamic therapy since, upon exposure to light, they pass from the intersystem to the triplet excited state, followed by the formation of singlet oxygen, which is a highly reactive species and mediates various oxidative processes [11]. In our previous study [2], the photodegradation rate of commercially available 1,4-DHPs was correlated to a series of descriptors, and the resulting model was used to design new congeners with high photostability. These drugs are L-type calcium channel blockers and are used for the treatment of cardiovascular disorders such as cardiac arrhythmias [7].

Herein, a QSPR model to predict the photostability of recently synthesized 1,4-DHPs is proposed. These compounds are characterized by a high sensitivity to light, resulting, in most cases, in an oxidation product in which the dihydropyridine ring undergoes aromatization, leading to the formation of other products at lower concentrations. After their introduction into the market, miscellaneous structural modifications on 1,4-DHPs resulted in efficient inhibitors of various calcium channels or in different pharmacological activities. Indeed, chemical modifications on the ester moiety of condensed 1,4-DHPs yielded up to thirty times more selectivity on T-type calcium channels compared to L-type, thus making them potentially useful in pain therapies [12,13].

Accordingly, among several derivatives recently synthesized and investigated for their calcium channel blocking activity, a series of variously substituted 1,4-DHP-based compounds has been selected for the elaboration of the QSPR model. In these compounds, the 1,4-DHP scaffold is included in a condensed ring system known as hexahydroquinoline. Chemical tailoring of hexahydroquinoline resulted in different types of calcium channel blockers with varying selectivity profiles. For example, the introduction of a pyridylmethyl moiety as the alkyl group into the ester function increased the blocking activity of this class of compounds (DHP series) against T-type calcium channels (Cav3.2) over L-type channels (Cav1.2) [14]. By focusing on the C-4 position of the main hexahydroquinoline scaffold, naphthyl and benzodioxole rings yielded potent and selective blockers of Cav1.2 (DA8) and Cav3.2 (DA1), respectively [15]. In the HM series, all compounds showed potent inhibitory effects on Cav1.2 currents. The HM8 molecule was found to be a selective blocker of Cav3.2 over Cav1.2 [12]. In the M and MD series, M3 was an effective and equipotent blocker of both L- and T-type calcium channels [13], and MD20 blocked Cav3.2 significantly [12].

Nimodipine, a first-generation 1,4-DHP-based L-type calcium channel blocker, was added to the selected compounds during model elaboration; thus, the calibration set had 20 molecules. Photodegradation was forced by exposing the drugs to a xenon lamp, in accordance with the International Conference on Harmonization (ICH) rules [16], and the degradation profile was monitored by spectrophotometric analysis. Over time, analytical methods have undergone considerable progress. In the past, it was necessary to use complex analytical methods to resolve a mixture of components and calculate the degradation kinetics. Now, new chemometric techniques allow the manipulation of spectrophotometric signals in order to obtain complete information for the description of a property of a class of drugs. These chemometric approaches that are easy to apply using modern algorithms have produced an empirical model derived from data that, from measurements, allows one or more properties of a system to be estimated. The evaluation of the degradation kinetics was carried out by applying the chemometric method Multivariate Curve Resolution-Alternating Least Squares (MCR-ALS) [3,17]. Results from MCR-ALS elaboration were confirmed by HPLC-DAD procedures. A dedicated software named PaDEL-Descriptor was used to calculate 1875 molecular descriptors [18]. PaDEL descriptors include 1D, 2D, and 3D molecular descriptors. Many of them could be correlated to a photodegradation process, especially to the oxidation reaction that occurs on the dihydropyridine ring of the tested compounds when exposed to light. For example, the AATSC5m PaDEL-Descriptor is related to the autocorrelation of a topological structure descriptor and denotes the distribution of properties along with the topological structure of compounds [19]. The Ordinary Least Squares (OLS) approach was used to select 17 descriptors and, subsequently, to elaborate the QSPR model. An independent Principal Component Analysis (PCA) of the original data was also applied to define the distribution of samples and molecular descriptors in the PC space. The QSPR model was able to correlate the photodegradation rate of nine new 1,4-DHPs with their chemical structure. Nicardipine, another first-generation 1,4-DHP drug, was added to this prediction set.

## 2. Materials and Methods

### 2.1. Instruments

Absorption spectra were registered in the range of 200–450 nm in a 10 mm quartz cell by means of a PerkinElmer Lambda 40P Spectrophotometer (PerkinElmer, Waltham, MA, USA) under the following conditions: scan rate: 1 nm s^−1^, time response: 1 s, and spectral band: 1 nm. Light exposure was simulated in a light cabinet, Suntest CPS+ (Heraeus, Milan, Italy), equipped with a Xenon lamp (Atlas Material Testing Technology, Mt Prospect, IL, USA), compliant with the ICH rules. The apparatus was fitted with an electronic device for irradiation, temperature measurement, and control inside the box. The system was able to closely simulate sunlight and appropriately select spectral regions between 300 and 800 nm through the interposition of filters. In particular, the application of the ID65 standard filter limits radiation to about 320 nm.

Chromatographic equipment consists of a HP 1100 pump fitted with a DAD G1315B (Agilent Technologies, Santa Clara, CA, USA) and a Rheodyne 7725 manual injector. The LC column was a C18 Gemini (Phenomenex, Bologna, Italy), 250 × 4.6 mm × 5 μm.

### 2.2. Software

The software UV Winlab^®^ 2.79.01 (PerkinElmer, Waltham, MA, USA) was used for spectral acquisition and elaboration. The PaDEL-Descriptor software version 2.21 (http://yapcwsoft.com/dd/padeldescriptor/ (accessed on 1 March 2023) using the Java language was used to calculate the molecular descriptors and fingerprints [20]. It consists of a library component that allows it to be easily integrated into quantitative structure–activity relationship software to provide the descriptor calculation feature and an interface component that allows it to be used as standalone software. All chemometric procedures were made with the Matlab^®^ computer environment software (Mathwork Inc., version 7, Natick, MA, USA). The drug concentration profile was calculated by applying the MCR algorithm to the spectral data to estimate the number of components, their spectra, and the rate constants (k) of the kinetic processes [17].

### 2.3. Chemicals

The work was applied to nineteen 1,4-DHPs synthesized in the Department of Pharmaceutical Chemistry (Pharmacy) of Hacettepe University, Ankara, Turkey, and to the first generation of the commercially available 1,4-DHP drug, Nimodipine (NIMO). In detail, the studied compounds include eleven 1,4-DHPs belonging to the DA series [15], three molecules of the DHP series [14], and five 1,4-DHPs of the HM series [12]. A prediction set was defined by using DA12, DHP5, DHP7, DHP9, DHP10, DHP12, and HM8, belonging to the same series used in the calibration set, and three other molecules belonging to classes of compounds different from those used for the construction of the chemometric model, that is, M3 to the M series [13], MD20 belonging to the MD series [12], and Nicardipine (NICA) as a first-generation drug. Briefly, the synthetic route adopted for the preparation of the compounds was as follows: substituted 1,3-cyclohexanedione, aldehyde derivative, and appropriate alkyl acetoacetate were heated in the presence of excess ammonium acetate. Absolute ethanol was used as a solvent. The precipitate formed after cooling the flask content or pouring it into ice water was further purified by washing with cold ethanol or crystallizing with ethanol-water to yield the target compounds. The chemical structures and the IUPAC name of all used molecules are listed in Table 1. Ethanol and acetonitrile of absolute grade were purchased by J.T. Baker (Amsterdam, The Netherlands).

### 2.4. Molecular Descriptors

The freely available PaDEL-Descriptor can calculate 1875 molecular descriptors and fingerprints [18]. Many types of molecular descriptors were developed [20,21], such as the number of carbon atoms, molecular weight, predictive values of LogP (XLogP, ALogP, etc.), properties calculated from two-dimensional (2D) structures (e.g., Eccentric Connectivity Index) and three-dimensional (3D) structures (e.g., charged partial surface area, CPSA), and properties based on quantum mechanics (orbital energies of the highest occupied molecular orbital (HOMO), lowest unoccupied molecular orbital (LUMO), etc.). Some additional descriptors and fingerprints were added, which include atom-type electrotopological state descriptors, McGowan volume, molecular linear free energy relation descriptors, ring counts, counts of chemical substructures identified by Laggner, and binary fingerprints and counts of chemical substructures identified by Klekota and Roth.

### 2.5. Standard Solutions

Standard solutions (about 20.0 μg mL^−1^) of each 1,4-DHP were prepared in ethanol to perform photodegradation experiments. More concentrated standard solutions (about 200.0 μg mL^−1^) in ethanol were prepared to define HPLC separations.

### 2.6. Experimental Conditions

The solution of each 1,4-DHP (20.0 μg mL^−1^) was directly light irradiated in a range between 300 and 800 nm by means of the ID65 standard filter; the irradiance power was fixed to 350 W m^−2^, corresponding to a light dose of 21 kJ min^−1^ m^−2^, at a constant temperature of 25 °C. The UV spectra were recorded just after the preparation and at the following interval times of light exposure: 1, 2, 3, 4, 5, 10, 15, 20, 25, 30, 40, 50, 60, 80, 100, 120, 150, 180, 210, 240, 270, and 300 min.

The mobile phase used in the HPLC analysis was water (A)–acetonitrile (B) pumped at a flow rate of 1 mL/min at room temperature. The solvents were filtered through a 0.45 μm membrane under vacuum. HPLC was run with 80% A and 10% B for 15 min in isocratic conditions. The injection volume was 20 μL. The UV–vis spectra were recorded between 200 and 450 nm, and the chromatographic profiles were registered at 236.8 nm with a reference at 400 nm. The chromatographic separation was carried out just after the preparation and at different interval times of light exposure up to 60 min and 600 min for NIMO and the other 1,4-DHPs, respectively.

## 3. Results

A series of 20 1,4-DHPs were collected to perform the QSPR study. As a first step, the molecules were subjected to forced photodegradation under the standard conditions described above. The MCR method was applied to the collected UV spectra to calculate the kinetic constant of the photodegradation process. The HPLC method was applied to confirm the formation of the photodegradation products. A large number of molecular descriptors were calculated, and 17 of them were selected to elaborate the QSPR model.

### 3.1. Photodegradation Studies

The sequence of the UV spectra during light irradiation was recorded for each 1,4-DHP solution (20.0 μg mL^−1^). According to our previous studies [12,13,14], a gradual decrease in the maximum peak in the zone 350–370 nm, which is a typical signal of the 1,4-DHP structure, and a contemporary increase in a new peak in the zone 260–280 nm, which is characteristic of the pyridinic structure, were observed for all the compounds. As an example, Figure 1A shows the sequence of the spectra recorded for DA9 and HM16. All the other collected spectral sequences are reported in the “Supplementary Materials” (Figures S1A–S20A).

**Figure 1:**
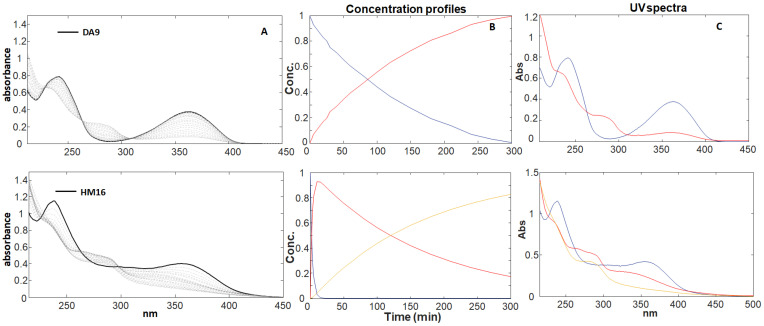
Photodegradation experiments of DA9 and HM16 at a concentration of 20.0 μg mL^−1^. (**A**) Spectral sequences and (**B**,**C**) concentration profiles and relative absorbance spectra, respectively, of the pure compounds (blue line) and the photoproducts (red and yellow lines) obtained from MCR elaboration.

The data matrix obtained from the collected spectra for each compound was analyzed by MCR-ALS, which aims to resolve the chemical contributions to the outcome of an experiment as described through a data matrix. The MCR method decomposes the experimental data matrix (D) into a reduced set of contributions of chemical species (in our study, 1,4-DHP and its degradation products) using a bilinear model. The number of components involved in the matrix D (chemical rank) can be estimated by PCA algorithms. The chemical rank assumes that the species contributing to the measured spectra have singular values larger than the other signal contributions, such as experimental or instrumental noise. Once the number of components is known, the ALS algorithm uses a series of constraints to optimize the MCR model. The application of constraints such as non-negativity, unimodality, and concentration closure allows one to optimize the results according to a chemical meaning. The quality and reliability of the multivariate resolution can be assessed using the explained variance (%R^2^) and the lack of fit (%). Wavelengths below 215 nm were discarded as preliminary selection, due to their high variability or instrumental noise. Therefore, the MCR processing was applied to spectral data between 215 and 450 for all 1,4-DHPs. Data processing shows the formation of a single photoproduct (PhP1) for the same molecules or traces of a second photoproduct (PhP2). Figure 1B and Figure 1C, respectively, showed the concentration profiles and the UV spectra of the pure compound and the relative photoproducts for DA9 (formation of one photoproduct) and HM16 (formation of two photoproducts). These graphs are elaborated for each compound and reported in “Supplementary Materials” (Figures S1B,C–S20B,C). The data from the photodegradation and MCR elaboration performed on the DHP series are published in our previous paper [14]. A first-order kinetic equation was calculated in all photodegradation experiments as follows:

ln [%1,4-DHP] = −k_1_ × t + 4.67

where %1,4-DHP is the percentage of residual absorbance, k_1_ is the photodegradation rate, t is the time (s), and 4.67 is the logarithm of initial absorbance (100%). In all experiments, the parameter lack of fit (% lof), which indicates the quality of the MCR results, was less than 7%, and the R^2^ was higher than 99.3%. The photodegradation rate of the studied drugs was also compared by measuring the parameter t_0.1_, which represents the time in which a 10% degradation was verified. Table 2 summarizes the kinetic parameters calculated for each 1,4-DHP. The data were collected from three replicate analyses for each sample, and very low variance was measured in all the cases.

In order to confirm the results obtained from MCR methods, a method for the determination of each compound in the presence of the photodegradation products was developed by HPLC-DAD. The chromatographic conditions were optimized to ensure the resolution of the mixture, showing close retention times. The best results were obtained by using a C18 stationary phase and a mobile phase consisting of water and acetonitrile pumped in isocratic elution as described in the “Section 2.6”. The used phase composition assured the complete solubilization of all the analytes. The calibration curves of the tested compounds were calculated by applying the HPLC procedure to five solutions of each compound, with concentrations ranging from 50.0 to 250.0 μg mL^−1^. The relative peak area was correlated to the respective drug concentration, showing correlation coefficients all over 0.98. As an example, the HPLC chromatogram of the NIMO solution showed a peak corresponding to the pure compound with a retention time (rt) of 1.169 min. The following calibration curve was calculated in this case:

[Ap] = 36.097 [C] + 4.281, R^2^ = 0.998

where Ap is the peak area (mAU*s) at the rt of 1.169 and C is the concentration of the sample (μg mL^−1^). Limit of detection (LOD) and limits of quantitation (LOQ) were also measured to be 1.56 μg mL^−1^ and 2.93–298.08 μg mL^−1^, respectively.

**Figure 2:**
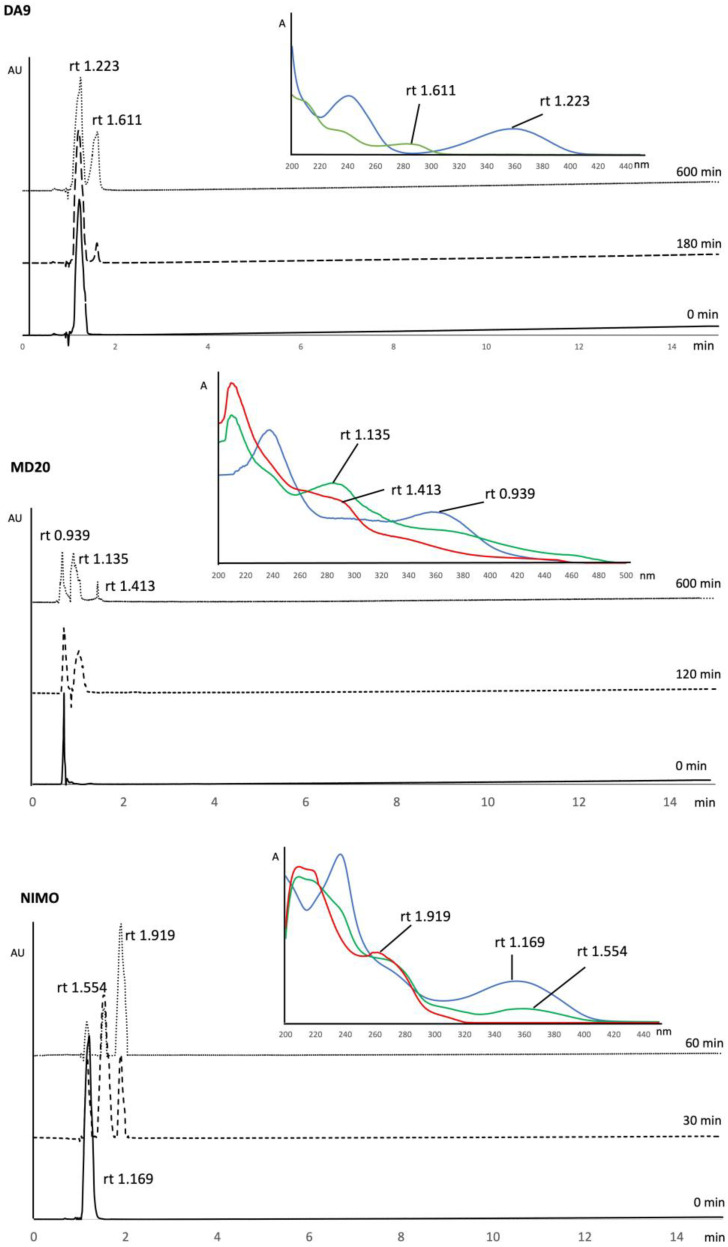
Sequence of the chromatograms performed for DA9, MD20, and NIMO and relative DAD absorbance spectra corresponding to the peaks.

In order to assess the photodegradation profile, a solution of each compound was prepared at a concentration value of about 200.0 μg mL^−1^ and exposed to light at different interval times. The presence of the photodegradation products was verified by the formation of one or two peaks in the chromatogram during the experiments. As an example, Figure 2 shows the sequence of the chromatograms performed for DA9, MD20, and NIMO. The DAD absorbance spectra of the drug peaks, reported in the same figure, were then used to confirm the identification of the pure compound and the photodegradation by-products. The peak purity of all the analytes was confirmed by the constancy of the DAD spectra along the single peaks.

### 3.2. Selection of Independent Variables for QSPR Method Elaboration

For each 1,4-DHP, 1875 molecular descriptors were calculated using PaDEL-Descriptor software version 2.21 and used to create the data matrix. The first optimization of this matrix was carried out by deleting the zero or constant variables. In this case, a matrix of 1427 descriptors was obtained. These new data were processed using MATLAB software (release R2021a, The MathWorks, Inc., MA, USA), in which the constant rate k of the degradation process was used as a dependent variable. The regression toolbox [22] includes four selection strategies (models of all subsets, direct selection, genetic algorithms, and sequential substitution remodeled), which can be coupled with a regression method. In our study, the selection of descriptors was a very complex procedure. Various algorithms have been tested, but none were able to define a group of variables that would provide satisfactory results in the subsequent prediction step. The selected algorithm was OLS, which provides the regression coefficients while minimizing the residual sum of squares (RSS). The aim of the method is to minimize the prediction error between the predicted and real values by taking into account the sum of squared errors instead of the errors as they are, because sometimes they can be negative or positive, and they could sum up to a nearly null value [23]. Specifically, the applied “Forward selection” allowed us to insert the variables sequentially within the model [24]. The first variable to enter into the equation is the one with the highest positive or negative correlation with the dependent variable. This variable is placed in the equation only if it satisfies the entry criterion. If the first variable has been included, the independent variable not present in the equation that has the highest partial correlation is considered the next one. The procedure ends when there are no more variables that satisfy the insertion criterion, and the determination of the more informative variables is defined by evaluating the regression coefficients of each variable. The processing performed selected 17 molecular descriptors as independent variables. Their values for each compound are summarized in Table 3. These descriptors included physicochemical parameters, 2D autocorrelations, and 3D spatial distributions. For example, the Ghose-Crippen-Viswanadhan octanol–water partition coefficient (ALogP) represents the logarithm of the partitioning coefficient between octanol and water used in determining both the pharmacokinetic and pharmacodynamic behaviors of a molecule. This parameter is calculated from the AlogP model, consisting of a regression equation based on the hydrophobicity contribution of 115 atom types, including C, H, O, N, S, Se, P, B, Si, and halogens [25]. The 2D autocorrelation encodes the structure of the molecules and the numerical properties assigned to atoms. In this series, the following parameters were selected: the autocorrelation descriptors as AATSC5m (centered Broto-Moreau autocorrelation—lag 5/weighted by mass), GATS5m (Geary autocorrelation—lag 5/weighted by mass), MATS4s (Moran autocorrelation—lag 4/weighted by I-state), and MATS5c (Moran autocorrelation—lag 5/weighted by charges); the electrotopological state atom-type descriptor as minHBd (minimum E-states for (strong) hydrogen bond donors), and minHBint7 (minimum E-state descriptors of strength for potential hydrogen bonds of path length 7); the largest chain descriptors as nAtomLC (number of atoms in the largest chain); the ring count descriptors as nFRing (fused ring count) and nT10HeteroRing (number of 10-membered rings (includes counts from fused rings) containing heteroatoms (N, O, P, S, or halogens)); and the ChiChain descriptor as SCH-6 (simple chain, order 6) and VCH-5 (Valence chain, order 5). The 3D correlations define the radial distribution function (RDF) in 3D space. Formally, the RDF of an ensemble of N atoms can be interpreted as the probability distribution to find an atom in a spherical volume of radius r. In this series, we used: RDF40m (040/weighted by relative mass), RDF45m (045/weighted by relative mass), RDF85m (085/weighted by relative mass), and RDF115e (115/weighted by relative Sanderson electronegativities). A 3D WHIM descriptor as E3m (3rd component accessibility directional WHIM index/weighted by relative mass) was also added [26].

**Figure 3:**
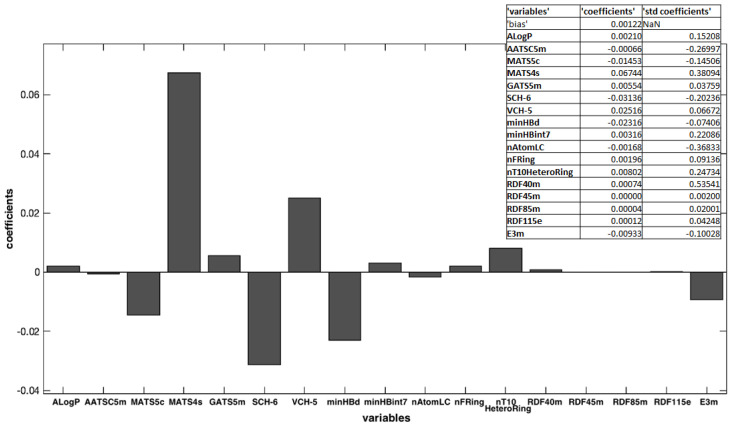
Weighted regression coefficients (Bw) calculated for all the x-variables used in OLS model elaboration.

Figure 3 shows the weighted regression coefficient (Bw) plot. In general, for all selected methods, such as fitness functions, the RMSECV and coefficient of determination (R^2^_cv_) are used in cross-validation.

**Figure 4:**
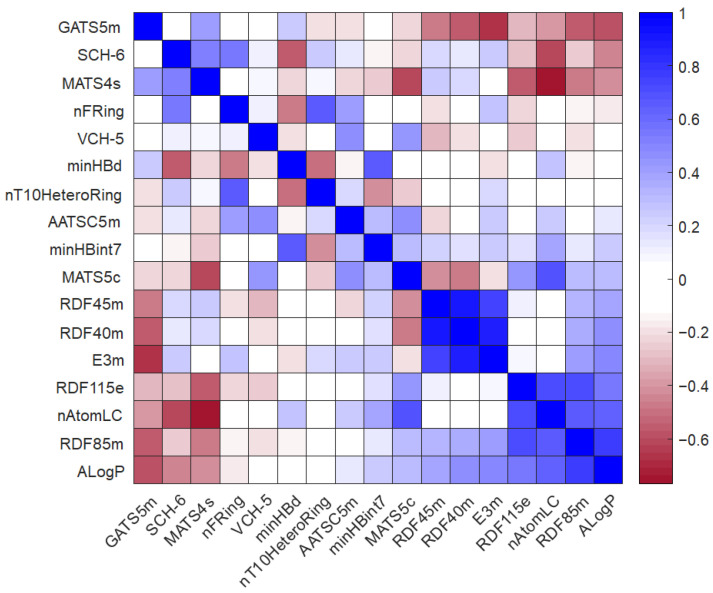
Pearson correlation coefficient (Rp^2^) calculated for the selected molecular descriptors.

In addition, the Pearson correlation heatmap of the model variables was calculated. It is depicted in Figure 4. This heatmap shows the relationship between the different molecular descriptors selected by calculating the Pearson coefficient (Rp^2^) [27]. In general, the overall correlation between the variables is low, with Rp^2^ values ranging from −0.4 to 0.4. However, some interesting positive relationships can be observed, as follows: RDF descriptors are highly correlated with each other, and a strong correlation was found when comparing nT10HeteroRing vs. nFRing and nAtomLC vs. MATS5c. In contrast, correlations with a negative sign were observed for the descriptor pairs E3m vs. GATS5m and nAtomLC vs. MATS4s.

### 3.3. QSPR Model Elaboration

**Figure 5:**
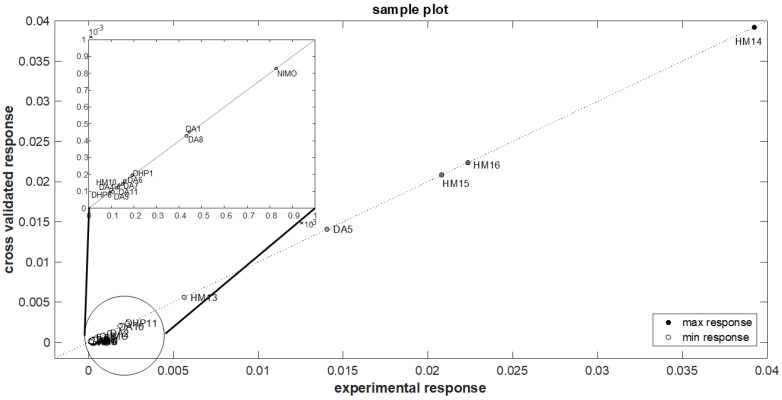
Sample distribution respect to the measured k values and the predicted ones of the optimized model.

The calibration set, based on the values of k (variable Y) as a function of the 17 molecular descriptors (variable X), was used to obtain the QSPR model by once again adopting the OLS algorithm. In a second step, a full-cross procedure was applied to validate the defined model, adopting a leave-one-out method. Satisfactory statistical results were obtained and reported in Table 4. Figure 5 shows the sample distribution in correlation with the measured k values and the predicted ones from the full cross-validation procedure. The OLS procedure carried out the following model equation:
K = 1.216 × 10^3^ + 2.100 × 10^−3^ ALogP − 6.597 × 10^−4^ AATSC5m − 1.453 × 10^−2^ MATS5c+ 6.744 × 10^−2^ MATS4s + 5.535 × 10^−3^ GATS5m − 3.136 × 10^−2^ SCH-6 + 2.516 × 10^−2^ VCH-5− 2.316 × 10^−2^ minHBd + 3.159 × 10^−3^ minHBint7 − 1.681 × 10^−3^ nAtomLC + 1.964 × 10^−3^ nFRing+ 8.021 × 10^−3^ nT10HeteroRing + 7.442 × 10^−4^ RDF40m + 3.947 × 10^−6^ RDF45m+ 4.102 × 10^−5^ RDF85m + 1.163 × 10^−5^ RDF115e − 9.327 × 10^−3^ E3m


**Figure 6:**
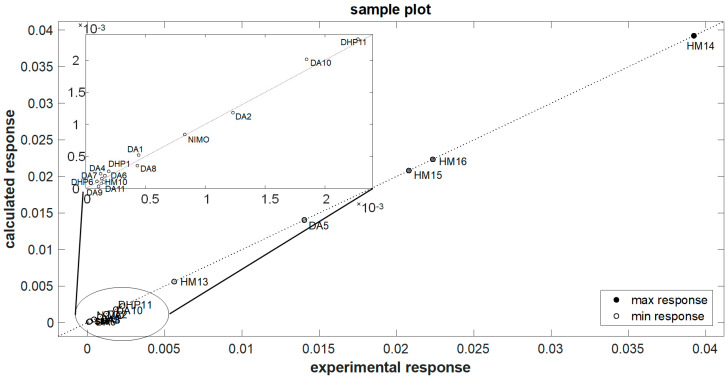
Application of the elaborated model to the external molecules of the prediction set.

**Figure 7:**
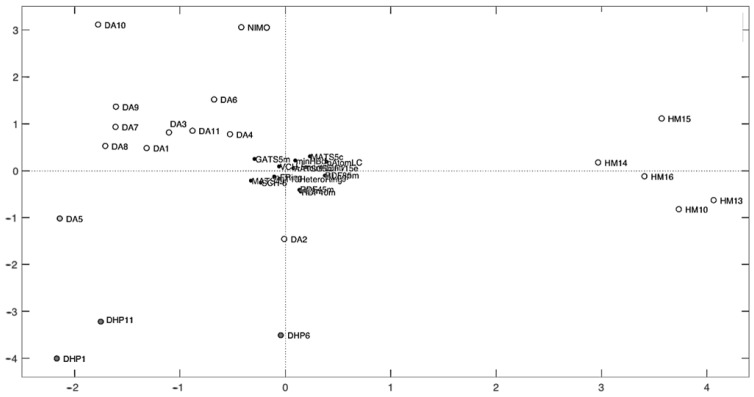
Bi-plot graph of scores and X-loadings.

As further validation, the defined model was applied to an external set of the molecules listed in the prediction set (Table 1), with very good results reported in Table 4 and depicted as a correlation between the experimental and calculated responses (Figure 6). In addition, an independent PCA on the original data, in which new variables were calculated as linear combinations of the old ones, was also performed. In these cases, the data were centered and scaled [2]. This elaboration allowed us to obtain two new matrices from the original input matrix (matrix X), represented by the scores (matrix of the sample information) and the loadings (matrix of the projections of the molecular descriptors in the PC space). The bi-plot graph shown in Figure 7 represents scores and X-loadings. Non-overlapping figures of scores and X-loadings are shown in the “Supplementary Materials” (Figures S21 and S22).

## 4. Discussion

In a general procedure of QSPR modeling, numerous molecular descriptors of each compound are calculated in the datasets, and a reliable model of the training dataset is constructed to predict a property from these calculated descriptors using classification or regression methods (e.g., multiple regression analysis, PLS regression, support vector machine (SVM), and random forest). As a last step, the performance of the elaborated model is evaluated by predicting the same properties of the compounds in the test dataset that are not used for model construction (prediction set).

Herein, a QSPR model was elaborated to correlate the photodegradation rate and the molecular structure of a small set of compounds belonging to different 1,4-DHP series. The presence of different functional groups on the same scaffold confers on the molecules different selectivities towards L- and T-type calcium channels as well as different sensitivity to light. Thus, the validated chemometric approach, by means of frequently used molecular descriptors, may provide reliable predictions regarding the light stability properties arising from the chemical structures of compounds.

First, the selected compounds in the ethanol solution were exposed to light under moderate irradiation conditions due to the high light sensitivity of some molecules compared to others. Some 1,4-DHPs completely degraded after only 15 min of exposure, whereas others still retained a residual percentage of 50% after 150 min, but at the end of the experiment (300 min), all molecules were completely degraded. In fact, t_0.1_ values ranged from 0.13 min for DA5 to 19.33 min for HM13. Data processing, carried out using the MCR procedure, highlighted the formation of PhP1 corresponding to the oxidation product in which the dihydropyridine ring undergoes aromatization for DA3, DA4, DA5, DA6, DA7, DA8, DA9, DA10, DA11, DA12, DHP1, DHP5, DHP6, DHP7, DHP9, DHP10, DHP11, DHP12, HM13, HM14, HM15, and NICA and a trace of PhP2 for DA1, DA2, HM8, HM10, HM16, MD20, M3, and NIMO. This procedure allowed us to define the value of the photodegradation rate (k_1_ and k_2_) for each compound, as reported in Table 2. The values of t_0.1_ were also calculated to compare the photodegradation process.

The results from chemometric methods were validated by HPLC-DAD analysis. The compounds were analyzed at different interval times, as described. During light exposure, the formation of secondary peaks was observed, and the correspondence with the absorption spectra obtained by MCR processing was verified. For example, Figure 2 shows the sequence of the chromatograms performed for DA9, MD20, and NIMO and the relative absorbance spectra corresponding to the chromatographic peaks. The obtained spectra for the pure compound and the by-products were very similar to those carried out from MCR procedures, despite the fact that the solvent of the mobile phase was different from the solvent used to prepare the standard solutions.

A dedicated software was used to calculate a large number of molecular descriptors that could be used to compare the properties of the different chemical structures. The most important variables were selected by using the OLS algorithm. The Toolbox includes the “Forward selection” approach, which allows starting from an empty set and adding variables sequentially to this set, one at a time. In each iteration, the criterion to select which variable to include is based on the minimization of RMSECV. In this approach, results can be biased by the first variables included in the selected set [24]. Among all the calculated descriptors, 17 were chosen as being responsible for significant changes in the molecular properties, with a RMSECV of 0.0002. This step is crucial and must be executed carefully to avoid both the exclusion of descriptors that bring useful information from the system and the indiscriminate use of a greater number of descriptors, which could increase random noise and reduce the robustness of the model. Figure 3 shows the Bw regression coefficient values. Some of them had a high Bw value and, of course, were most important in the model building. Other ones showed a small coefficient, and their influence may be negligible. Moreover, the variables with a positive regression coefficient were directly proportional to the response, and, on the contrary, the other with a negative coefficient were indirectly proportional. In this case, the most important variables were MATS4s, SCH-6, VCH-5, minHBd, and MATS5s, all belonging to the 2D autocorrelation descriptors. Moreover, MATS4s and VCH-5 were directly correlated to the k values: molecules that have high positive values for these descriptors showed a high value of k and negatively affected the photodegradation process; thus, high values of these descriptors make the molecules less light-stable. On the contrary, SCH-6, minHBd, and MATS5s were inversely correlated to the k values, that is, high values of these molecular descriptors lead to greater stability of the molecules. The importance of these descriptors can be due to their influence on the oxidation process of the tested molecules. In fact, 2D autocorrelation descriptors are related to the spatial distribution of a generic molecular property in the molecular structure space and measure the strength of a relationship between atoms at a predefined distance. They are calculated by using various molecular properties that can be represented at the atomic or molecular surface level [6]. For example, MATS4s defines the distribution of physicochemical properties along the topological surfaces of the molecules as a positive coefficient, and it is weighted by Sanderson electronegativities [28]. According to Miranda-Quintana et al., the oxidation potential of a molecule increases as its electronegativity increases and also increases as its electronegativity in its oxidized state increases [29]. Thus, high values of these autocorrelation descriptors increase the k value and increase the photodegradation rate of the molecules.

In a second step, the QSPR model was elaborated to correlate photodegradation rate and molecular descriptors. The model was validated by the fully cross method, adopting a leave-one-out procedure, and as reported in Table 4, satisfactory statistical results were carried out with a relative error percentage of 0.090% associated with an optimized number of eight PC. This multivariate regression allowed us to obtain the equation reported above, which defines the influence of explanatory variables on a dependent variable [30]. The model calculates the partial regression coefficients, which measure, in our case, the contribution of each descriptor as the value of k varies. From a statistical point of view, the calculated equation represents the mathematical formalization of the associative link between k and the previously selected molecular descriptors. The sign of the regression coefficient indicates the “direction” of the relationship: the positive sign indicates a concordance between the variables (an increase in x corresponds to an increase in y), and the negative sign indicates a discordance (an increase in x corresponds to a decrease in y). The absolute value of b indicates the “degree” of the relationship (the larger the value of b, the more the variable x influences the variable y).

In addition, PCA explorative analysis allows us to obtain a bi-plot graph of scores and loadings showing the distribution of the molecules with respect to the descriptors and giving 28.45% of the explained variance (EV) for PC1 and 21.94% EV for PC2 (Figure 7 or Figures S21 and S22). Score plots allowed us to rapidly locate similar objects: similar-structure molecules positioned themselves close together; in fact, three groups of molecules can be identified in the score plot corresponding to the different classes of compounds. DA series were close in the upper left quadrant, whereas HM series were close in the right quadrant, and DHP series were distributed in the lower left quadrant. This demonstrates how the presence of a distinct halogen group on the benzene ring affects the various molecular properties. Strongly correlated variables will have approximately the same weight value when they are positively correlated, and in a loadings plot, they will appear near each other, while negatively correlated variables will appear diagonally opposite to each other. Thus, drugs placed in the right-hand quadrant of the score plot had a large value for the variables located to the right of the loading plot and a small value for the variables to the left of the loading plot. Among the most important descriptors, the compound belonging to the HM series had large values of minHBd and MATS5s and small values of MATS4s and SCH-6. On the contrary, DA and DHP compounds had large values of MATS4s, SCH-6, and VCH-5 and small values of minHBd and MATS5s.

An independent validation was performed by applying the defined model to an external set of the molecules listed in the prediction set (Table 1). These compounds were subjected to photodegradation under the same stress conditions adopted for the calibration samples, and the k values were calculated by MCR analysis. As reported in Figure 6 and Table 4, the photodegradation rate of these molecules was predicted with satisfactory results and a relative error percentage of 5.03%. Based on these results and comparing the structures of the different molecules, it can be assumed that the presence of two NO groups on the pyridine ring influences the stability of molecules, making them more sensitive to light. These molecules showed indeed lower t_0.1_ values. The presence of halogens, instead, confers high stability.

However, reaching definitive conclusions may not be straightforward considering that the scaffold of the studied compounds is variously functionalized. Consequently, developing a QSPR prediction model capable of correlating the three-dimensional chemical features of each compound with its sensitivity to light might be an invaluable tool in better understanding the pharmaceutical properties of this class of compounds.

## 5. Conclusions

The combined use of QSPR analysis and multivariate methods represents an innovative and promising approach in the analytical field. QSPR studies allow for the correlation of the structural features of a molecule with a chemical or physical property. On the other hand, chemometric methods can help define a simple and easy analytical procedure able to build a QSPR elaboration. Herein, a QSPR model correlating the sensitivity to light of recently synthesized 1,4-DHP compounds with molecular descriptors was elaborated with satisfactory results, demonstrating a very good ability in the prediction of the stability of congeneric drugs not used in the model construction.

## Figures and Tables

**Table 1:** Chemical structures and IUPAC names of the selected compounds.

	***Calibration Set***		
	**Compound**	**Chemical Structure**	**IUPAC Name**
1	DA1	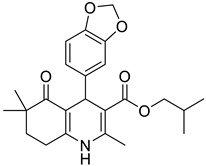	Isobutyl 4-(benzo[*d*][1,3]dioxol-5-yl)-2,6,6-trimethyl-5-oxo-1,4,5,6,7,8-hexahydroquinoline-3-carboxylate
2	DA2	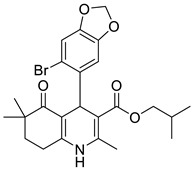	Isobutyl 4-(6-bromobenzo[*d*][1,3]dioxol-5-yl)-2,6,6-trimethyl-5-oxo-1,4,5,6,7,8-hexahydroquinoline-3-carboxylate
3	DA3	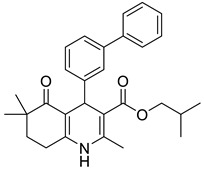	Isobutyl 4-([1,1′-biphenyl]-3-yl)-2,6,6-trimethyl-5-oxo-1,4,5,6,7,8-hexahydroquinoline-3-carboxylate
4	DA4	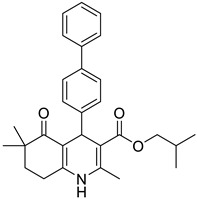	Isobutyl 4-([1,1′-biphenyl]-4-yl)-2,6,6-trimethyl-5-oxo-1,4,5,6,7,8-hexahydroquinoline-3-carboxylate
5	DA5	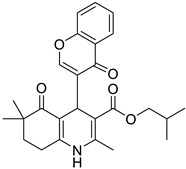	Isobutyl 2,6,6-trimethyl-5-oxo-4-(4-oxo-4*H*-chromen-3-yl)-1,4,5,6,7,8-hexahydroquinoline-3-carboxylate
6	DA6	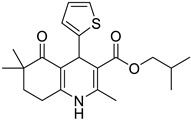	Isobutyl 2,6,6-trimethyl-5-oxo-4-(thiophen-2-yl)-1,4,5,6,7,8-hexahydroquinoline-3-carboxylate
7	DA7	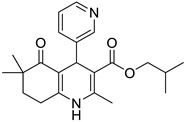	Isobutyl 2,6,6-trimethyl-5-oxo-4-(pyridin-3-yl)-1,4,5,6,7,8-hexahydroquinoline-3-carboxylate
8	DA8	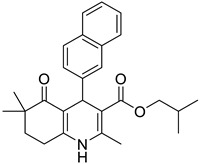	Isobutyl 2,6,6-trimethyl-4-(naphthalen-2-yl)-5-oxo-1,4,5,6,7,8-hexahydroquinoline-3-carboxylate
9	DA9	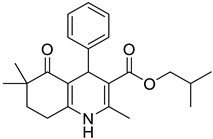	Isobutyl 2,6,6-trimethyl-5-oxo-4-phenyl-1,4,5,6,7,8-hexahydroquinoline-3-carboxylate
10	DA10	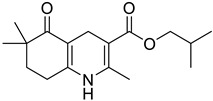	Isobutyl 2,6,6-trimethyl-5-oxo-1,4,5,6,7,8-hexahydroquinoline-3-carboxylate
11	DA11	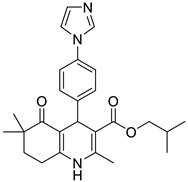	Isobutyl 4-(4-(1*H*-imidazol-1-yl)phenyl)-2,6,6-trimethyl-5-oxo-1,4,5,6,7,8-hexahydroquinoline-3-carboxylate
12	DHP1	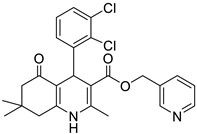	Pyridin-3-ylmethyl 4-(2,3-dichlorophenyl)-2,7,7-trimethyl-5-oxo-1,4,5,6,7,8-hexahydroquinoline-3-carboxylate
13	DHP6	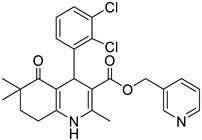	Pyridin-3-ylmethyl 4-(2,3-dichlorophenyl)-2,6,6-trimethyl-5-oxo-1,4,5,6,7,8-hexahydroquinoline-3-carboxylate
14	DHP11	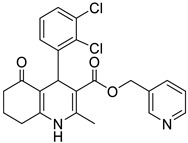	Pyridin-3-ylmethyl 4-(2,3-dichlorophenyl)-2-methyl-5-oxo-1,4,5,6,7,8-hexahydroquinoline-3-carboxylate
15	HM10	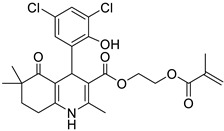	2-(Methacryloyloxy)ethyl 4-(3,5-dichloro-2-hydroxyphenyl)-2,6,6-trimethyl-5-oxo-1,4,5,6,7,8-hexahydroquinoline-3-carboxylate
16	HM13	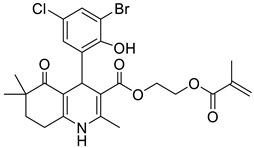	2-(Methacryloyloxy)ethyl 4-(3-bromo-5-chloro-2-hydroxyphenyl)-2,6,6-trimethyl-5-oxo-1,4,5,6,7,8-hexahydroquinoline-3-carboxylate
17	HM14	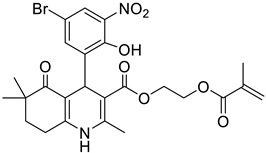	2-(Methacryloyloxy)ethyl 4-(5-bromo-2-hydroxy-3-nitrophenyl)-2,6,6-trimethyl-5-oxo-1,4,5,6,7,8-hexahydroquinoline-3-carboxylate
18	HM15	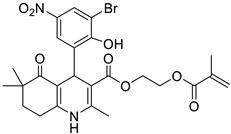	2-(Methacryloyloxy)ethyl 4-(3-bromo-2-hydroxy-5-nitrophenyl)-2,6,6-trimethyl-5-oxo-1,4,5,6,7,8-hexahydroquinoline-3-carboxylate
19	HM16	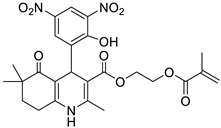	2-(Methacryloyloxy)ethyl 4-(2-hydroxy-3,5-dinitrophenyl)-2,6,6-trimethyl-5-oxo-1,4,5,6,7,8-hexahydroquinoline-3-carboxylate
20	NIMO	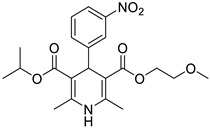	3-isopropyl 5-(2-methoxyethyl) 2,6-dimethyl-4-(3-nitrophenyl)-1,4-dihydropyridine-3,5-dicarboxylate
	***Prediction Set***		
	**Compound**	**Chemical Structure**	**IUPAC Name**
1	DA12	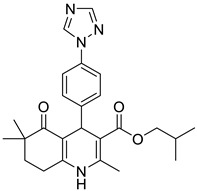	Isobutyl 4-(4-(1*H*-1,2,4-triazol-1-yl)phenyl)-2,6,6-trimethyl-5-oxo-1,4,5,6,7,8-hexahydroquinoline-3-carboxylate
2	DHP5	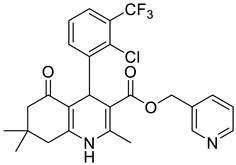	Pyridin-3-ylmethyl 4-(2-chloro-3-(trifluoromethyl)phenyl)-2,7,7-trimethyl-5-oxo-1,4,5,6,7,8-hexahydroquinoline-3-carboxylate
3	DHP7	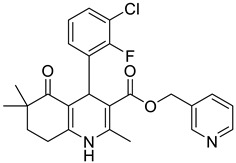	Pyridin-3-ylmethyl 4-(3-chloro-2-fluorophenyl)-2,6,6-trimethyl-5-oxo-1,4,5,6,7,8-hexahydroquinoline-3-carboxylate
4	DHP9	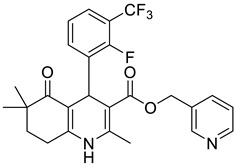	Pyridin-3-ylmethyl 4-(2-fluoro-3-(trifluoromethyl)phenyl)-2,6,6-trimethyl-5-oxo-1,4,5,6,7,8-hexahydroquinoline-3-carboxylate
5	DHP10	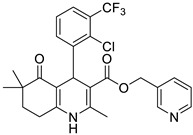	Pyridin-3-ylmethyl 4-(2-chloro-3-(trifluoromethyl)phenyl)-2,6,6-trimethyl-5-oxo-1,4,5,6,7,8-hexahydroquinoline-3-carboxylate
6	DHP12	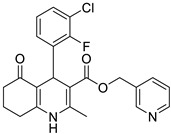	Pyridin-3-ylmethyl 4-(3-chloro-2-fluorophenyl)-2-methyl-5-oxo-1,4,5,6,7,8-hexahydroquinoline-3-carboxylate
7	HM8	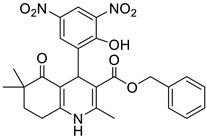	Benzyl 4-(2-hydroxy-3,5-dinitrophenyl)-2,6,6-trimethyl-5-oxo-1,4,5,6,7,8-hexahydroquinoline-3-carboxylate
8	M3	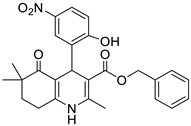	Benzyl 4-(2-hydroxy-5-nitrophenyl)-2,6,6-trimethyl-5-oxo-1,4,5,6,7,8-hexahydroquinoline-3-carboxylate
9	MD20	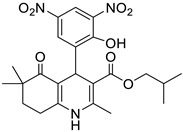	Isobutyl 4-(2-hydroxy-3,5-dinitrophenyl)-2,6,6-trimethyl-5-oxo-1,4,5,6,7,8-hexahydroquinoline-3-carboxylate
10	NICA	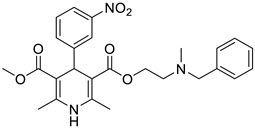	3-(2-(benzyl(methyl)amino)ethyl) 5-methyl 2,6-dimethyl-4-(3-nitrophenyl)-1,4-dihydropyridine-3,5-dicarboxylate

**Table 2:** Kinetics parameters calculated for each 1,4-DHP.

Drugs	k_1_ (×10^−4^)	SD (±) k_1_ (×10^−4^)	k_2_ (×10^−4^)	SD (±) k_2_ (×10^−4^)	R^2^	t_0.1_ (min)	% lof
DA1	4.435	0.107	0.389	0.013	99.89	4.134	3.281
DA2	12.31	0.494	1.771	0.060	99.73	1.491	5.153
DA3	1.217	0.022	-	-	99.93	15.064	2.693
DA4	1.226	0.020	-	-	99.77	14.954	4.758
DA5	140.4	6.183	-	-	99.17	0.131	6.085
DA6	1.597	0.071	-	-	99.57	11.483	6.553
DA7	1.345	0.012	-	-	99.96	13.633	1.963
DA8	4.322	0.091	-	-	99.96	4.242	2.105
DA9	1.061	0.005	-	-	99.99	17.273	0.919
DA10	18.48	0.775	-	-	99.36	0.992	6.981
DA11	1.315	0.016	-	-	99.94	13.939	2.406
DA12	1.389	0.009	-	-	99.99	13.204	0.999
DHP1	392.4	7.800	-	-	99.95	1.171	1.910
DHP5	376.3	4.900	-	-	99.89	1.220	1.730
DHP6	208.0	5.000	-	-	99.87	2.200	2.550
DHP7	91.00	1.900	-	-	99.67	5.030	1.751
DHP9	143.3	4.300	-	-	99.83	3.190	2.392
DHP10	222.3	8.100	-	-	99.58	2.060	2.251
DHP11	223.5	8.900	-	-	99.64	2.051	1.751
DHP12	81.30	1.800	-	-	99.78	5.631	1.232
HM8	101.0	4.798	2.354	0.070	99.71	0.182	5.341
HM10	1.918	0.060	0.752	0.032	99.94	9.562	2.456
HM13	0.950	0.300	-	-	99.94	19.329	2.512
HM14	22.77	0.501	-	-	99.97	0.805	3.643
HM15	1.494	0.039	-	-	99.32	12.274	6.263
HM16	56.28	1.262	0.781	0.013	99.95	0.326	2.199
MD20	78.12	2.442	2.335	0.050	99.84	0.235	4.035
M3	2.642	0.030	1.271	0.017	99.97	6.940	1.836
NICA	6.044	0.211	-	-	99.84	3.030	3.941
NIMO	8.290	0.494	8.290	0.762	99.98	2.211	1.501

**Table 3:** Molecular descriptors values selected as independent variables.

Compound	ALogP	AATSC5m	MATS5c	MATS4s	GATS5m	SCH-6	VCH-5	minHBd	minHBint7	nAtomLC	nFRing	nT10HeteroRing	RDF40m	RDF45m	RDF85m	RDF115e	E3m
*Calibration set*																	
DA1	0.895	0.5349	−0.0763	−0.091	0.960	0.328	0.029	0.277	1.541	7	2	1	12.672	13.082	6.3882	5.3440	0.219
DA2	1.784	12.455	−0.1343	−0.092	0.928	0.316	0.029	0.266	1.500	7	2	1	23.441	14.137	6.3882	5.3440	0.468
DA3	1.198	0.6475	−0.1391	−0.070	0.995	0.279	0	0.252	0	7	1	1	12.991	12.828	8.4192	11.175	0.108
DA4	1.198	0.3788	−0.1377	−0.071	1.005	0.279	0	0.255	0	7	1	1	12.987	11.390	10.488	15.520	0.172
DA5	0.531	1.0708	−0.3468	−0.070	0.966	0.232	0	0.287	0	7	2	2	18.319	12.670	5.6093	7.2459	0.244
DA6	1.721	7.3378	−0.0287	−0.071	0.914	0.177	0.118	0.262	0	7	1	1	14.711	8.9512	5.1640	5.3099	0.201
DA7	1.198	1.0262	−0.2355	−0.079	0.991	0.196	0	0.279	0	7	1	1	11.716	11.609	4.6429	5.3100	0.179
DA8	1.198	0.7416	−0.1399	−0.074	0.996	0.245	0	0.257	0	7	2	1	11.954	11.300	6.8782	5.4597	0.190
DA9	1.198	1.1070	−0.1436	−0.070	1.001	0.196	0	0.269	0	7	1	1	11.656	11.066	4.8526	5.3092	0.129
DA10	1.588	−2.2961	−0.2223	−0.119	1.087	0.104	0	0.307	0	7	1	1	8.6260	4.6878	3.0658	5.3097	0.051
DA11	1.211	−0.0733	−0.0964	−0.074	0.997	0.260	0.039	0.264	0	7	1	1	12.545	14.596	10.913	9.0331	0.154
DHP1	2.151	−6.9717	−0.4033	0.094	1.013	0.264	0	0.248	0.183	4	1	1	31.851	23.834	9.9394	4.3322	0.366
DHP6	2.113	1.0891	−0.3044	−0.084	0.869	0.264	0	0.261	0.191	4	1	1	33.429	25.382	8.1966	7.1145	0.364
DHP11	0.884	−3.0051	−0.3548	−0.033	0.939	0.284	0	0.288	0.215	4	1	1	31.568	23.373	7.8238	4.0094	0.386
HM10	2.728	−0.5443	−0.0922	−0.184	0.850	0.149	0	0.268	0.121	11	1	1	26.156	19.072	14.842	13.939	0.379
HM13	2.872	−3.5382	−0.0939	−0.181	0.791	0.149	0	0.266	0.163	11	1	1	22.243	15.955	22.571	11.192	0.322
HM14	2.788	7.1796	−0.0891	−0.092	0.941	0.149	0	0.308	1.532	11	1	1	21.555	19.234	13.523	11.140	0.306
HM15	2.788	2.2566	−0.1016	−0.183	0.988	0.149	0	0.291	1.532	11	1	1	20.069	13.500	19.511	14.199	0.256
HM16	2.559	1.2276	−0.1192	−0.119	0.880	0.149	0	0.336	1.648	11	1	1	23.994	21.070	11.162	14.199	0.323
NIMO	0.727	−4.1293	−0.1769	−0.095	1.057	0.129	0	0.390	1.906	7	0	0	16.456	15.744	4.2296	6.2738	0.111
*Prediction set*																	
DA12	1.211	−0.4667	−0.1001	−0.078	0.997	0.260	0.029	0.269	0	7	1	1	12.708	12.200	10.202	14.360	0.175
DHP5	2.505	−5.7584	−0.2736	0.013	1.016	0.264	0	0.285	0	4	1	1	35.806	22.011	12.323	5.1042	0.271
DHP7	1.849	0.5783	−0.3282	−0.106	0.882	0.264	0	0.292	0.092	4	1	1	22.168	18.126	8.0499	7.1145	0.342
DHP9	2.203	1.4613	−0.2254	0.062	0.873	0.264	0	0.329	0	4	1	1	17.679	16.303	10.610	7.8853	0.253
DHP10	2.467	1.9612	−0.2013	−0.082	0.861	0.264	0	0.298	0	4	1	1	37.457	23.559	10.756	7.8853	0.258
DHP12	0.619	−3.2561	−0.3772	−0.087	0.954	0.284	0	0.319	0.105	4	1	1	21.045	16.118	7.6773	4.0094	0.359
HM8	1.485	4.3519	−0.2163	−0.054	0.832	0.251	0	0.321	0	4	1	1	24.438	19.468	13.238	10.310	0.343
M3	0.825	3.5196	−0.2307	−0.114	0.874	0.264	0	0.302	0	4	1	1	17.510	12.141	7.5004	6.4756	0.302
MD20	2.058	3.0868	−0.2009	−0.043	0.838	0.149	0	0.316	0	7	1	1	22.172	15.548	8.1647	5.3099	0.418
NICA	0.906	−3.4479	−0.2082	−0.037	1.091	0.231	0	0.364	0.741	8	0	0	19.970	15.665	9.4702	10.748	0.115

**Table 4:** Statistical results obtained after application of full cross-validation procedure.

Compound	k (×10^−3^)	k (×10^−3^)	Error %
	Experimental Value	Predicted Value	
*Calibration set*			
DA1	0.443	0.452	1.878
DA2	1.231	1.226	−0.400
DA3	0.122	0.120	−1.215
DA4	0.123	0.129	4.884
DA5	14.042	14.043	0.007
DA6	0.160	0.161	0.632
DA7	0.134	0.136	0.788
DA8	0.432	0.429	−0.788
DA9	0.106	0.097	−9.039
DA10	1.848	1.852	0.221
DA11	0.132	0.126	−4.333
DHP1	39.240	39.239	−0.002
DHP6	20.800	20.801	0.006
DHP11	22.350	22.351	0.005
HM10	0.192	0.197	2.814
HM13	0.095	0.095	0.299
HM14	2.277	2.292	0.623
HM15	0.149	0.143	−4.165
HM16	5.628	5.616	−0.221
NIMO	0.829	0.830	0.118
*Prediction set*			
DA12	0.139	0.135	−2.704
DHP5	37.630	35.454	−5.783
DHP7	9.100	9.683	6.403
DHP9	14.330	15.072	5.179
DHP10	22.230	22.264	0.154
DHP12	8.130	8.770	7.876
HM8	10.099	10.365	2.635
M3	0.264	0.228	−13.662
MD20	7.812	7.978	2.123
NICA	0.604	0.640	5.813

## Data Availability

Almost all the data presented in this study are available in the Supplementary Materials or on request from the corresponding author.

## Supplementary Materials

The following supporting information can be downloaded at: https://www.mdpi.com/article/10.3390/pharmaceutics16020206/s1, Figures S1–S20. Photodegradation experiments of each tested compound at a concentration of 20.0 μg mL^−1^. (A) Spectral sequences, (B) concentration profiles of the pure compounds and the photoproducts obtained from MCR elaboration, and (C) relative absorbance spectra. Figure S1: DA1; Figure S2: DA2; Figure S3: DA3; Figure S4: DA4; Figure S5: DA5; Figure S6: DA6; Figure S7: DA7; Figure S8: DA8; Figure S9: DA10; Figure S10: DA11; Figure S11: DA12; Figure S12: HM8; Figure S13: HM10; Figure S14: HM13; Figure S15: HM14; Figure S16: HM15; Figure S17: MD20; Figure S18: M3; Figure S19: NIMO; Figure S20: NICA; Figure S21: Scores plot; Figure S22: X- and Y-loadings plot.
