# Supplementary material for: Multivariate Approaches in Quantitative Structure–Property Relationships Study for the Photostability Assessment of 1,4-Dihydropyridine Derivatives
Source: Pharmaceutics. 2024 Jan 31;16(2):206. doi: 10.3390/pharmaceutics16020206 (PMC10891640; doi:10.3390/pharmaceutics16020206)

Figure S1-20. Photodegradation experiments of each tested compound at a concentration of 20.0 µg mL^{-1}. A: Spectral sequences; B: concentration profiles of the pure compounds and the photoproducts obtained from MCR elaboration and C: relative absorbance spectra.

Figure S1. DA1.

A

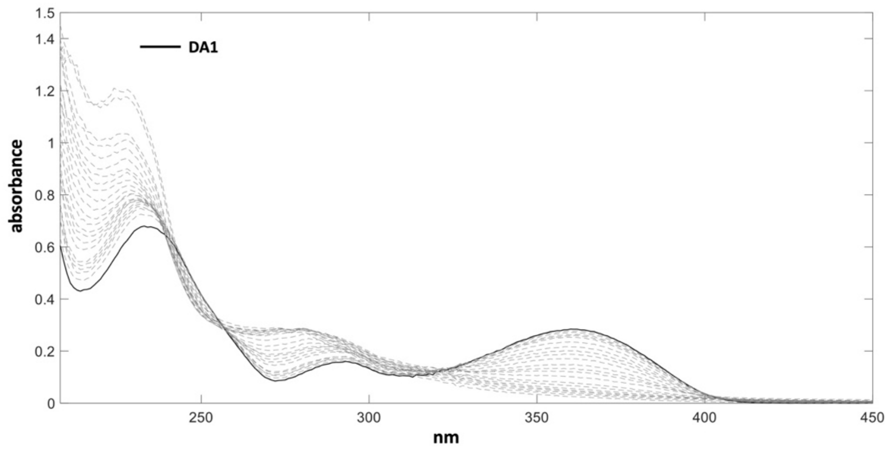

B

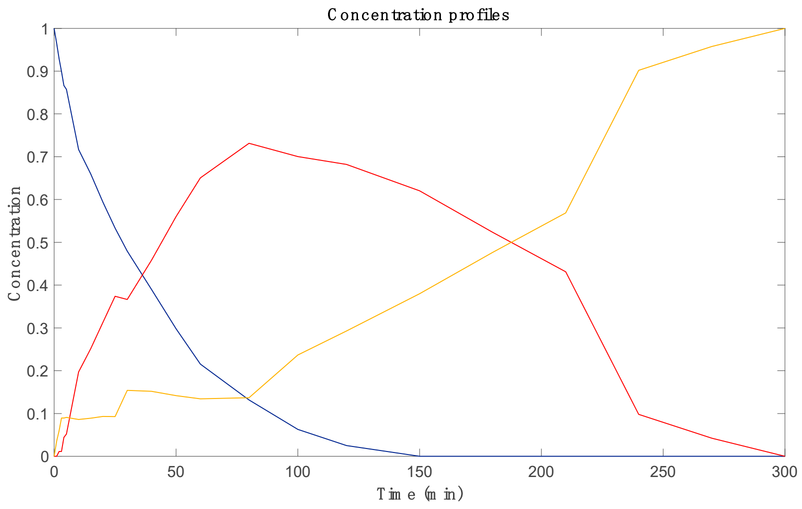

C

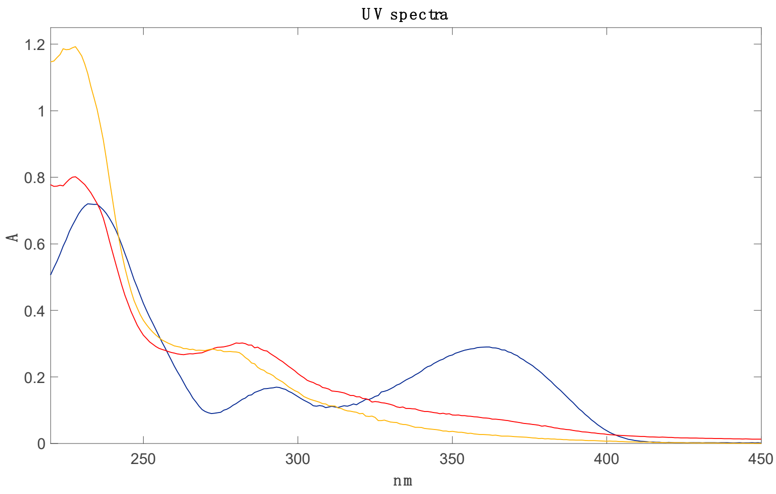

Figure S2. DA2.

A

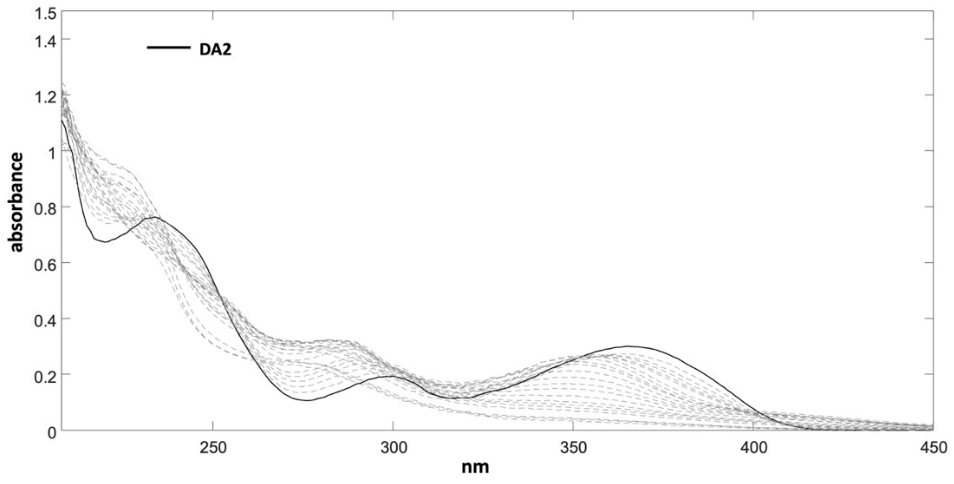

B

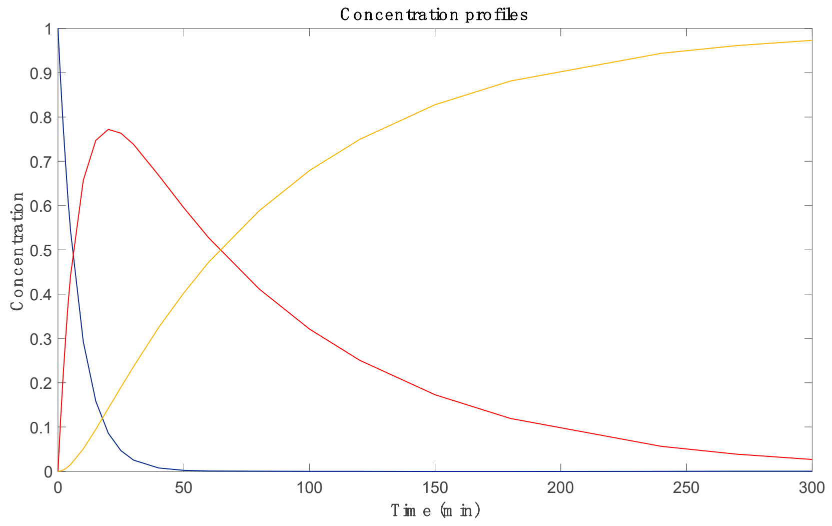

C

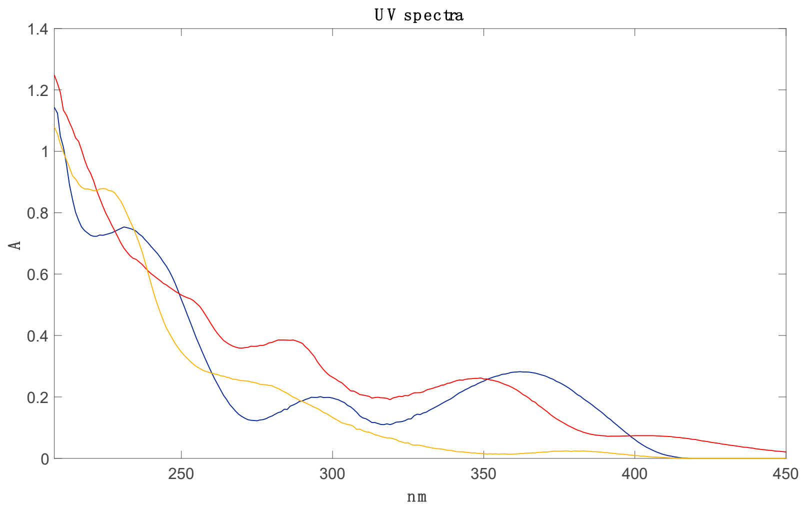

Figure S3. DA3.

A

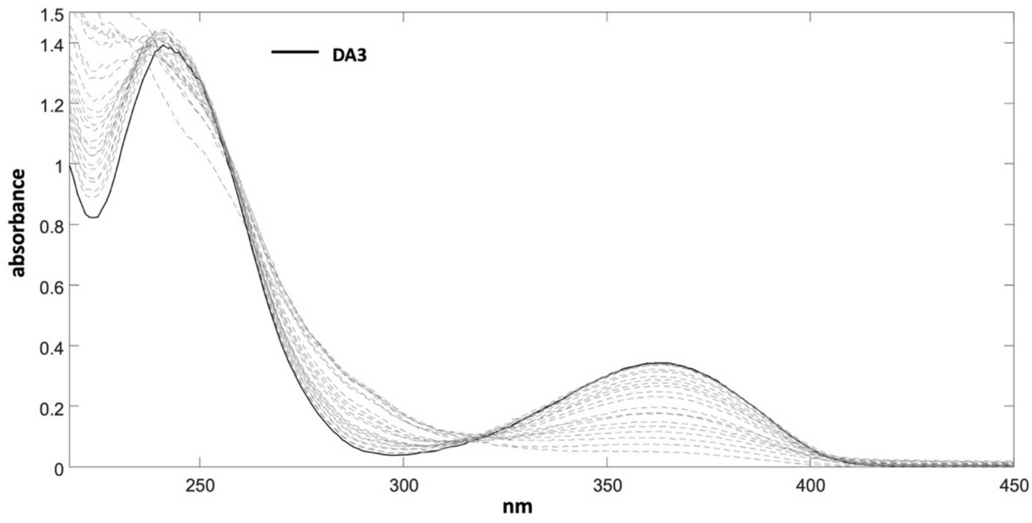

B

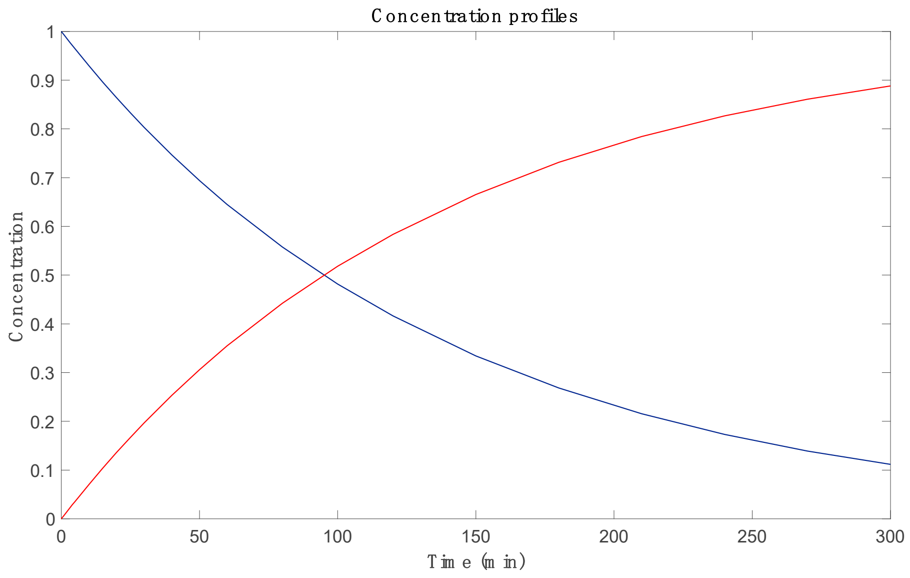

C

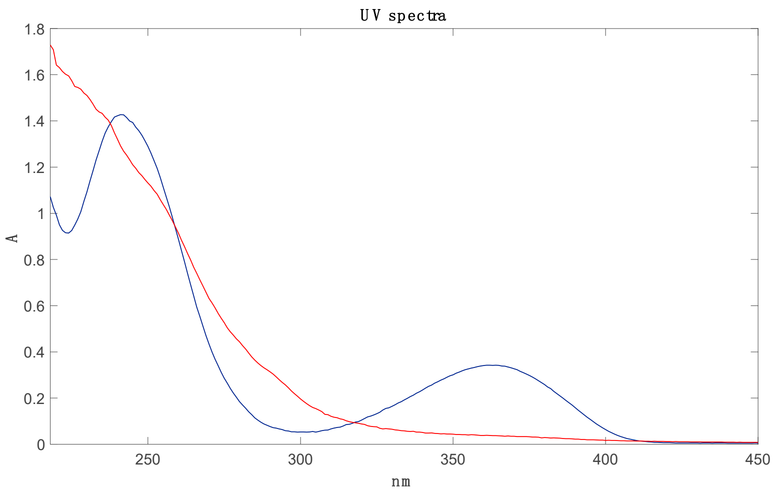

Figure S4. DA4.

A

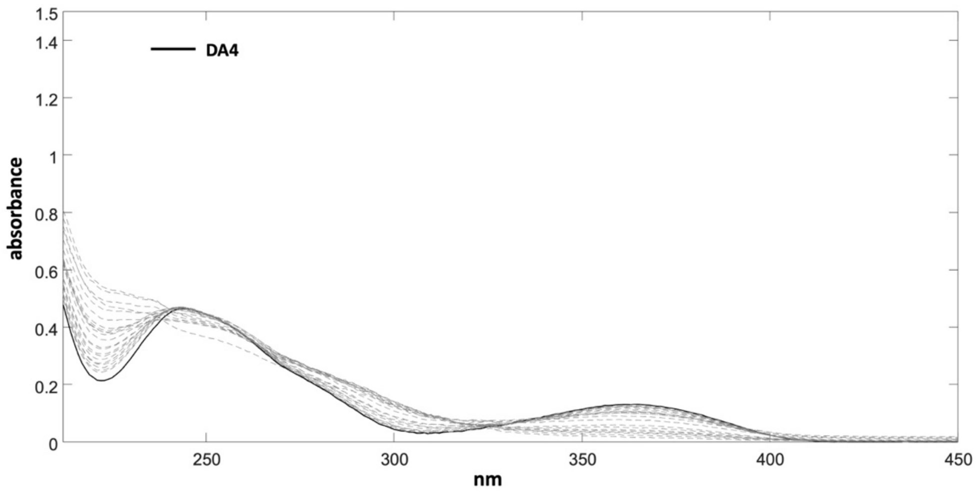

B

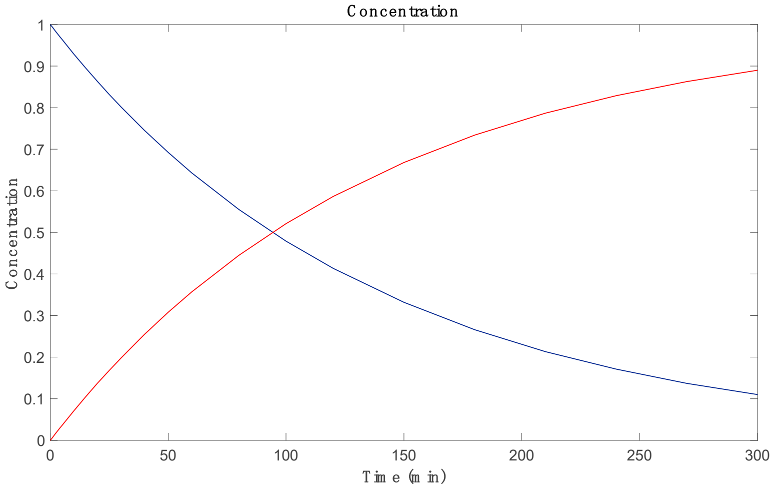

C

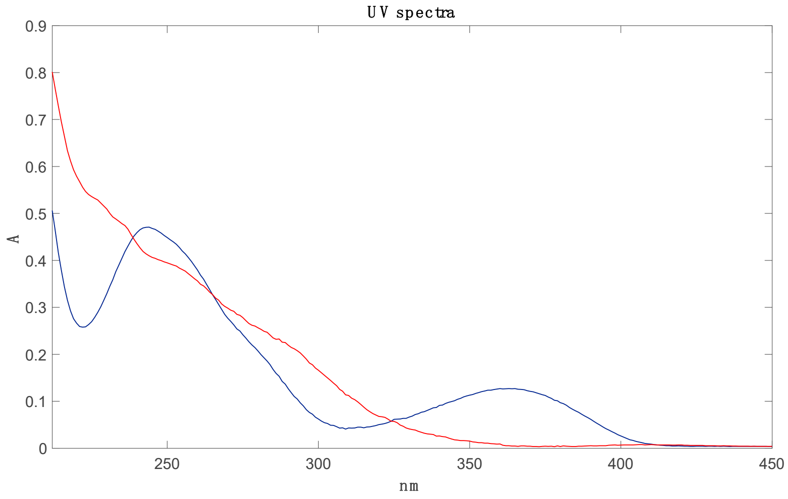

Figure S5. DA5.

A

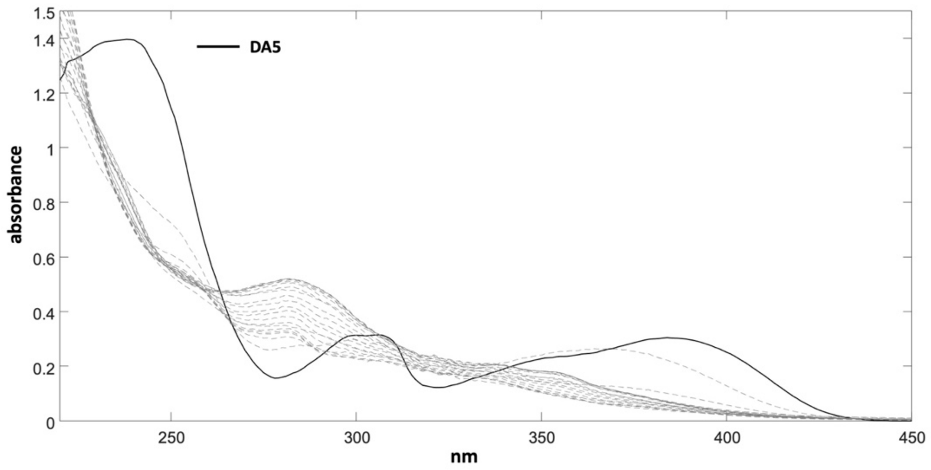

B

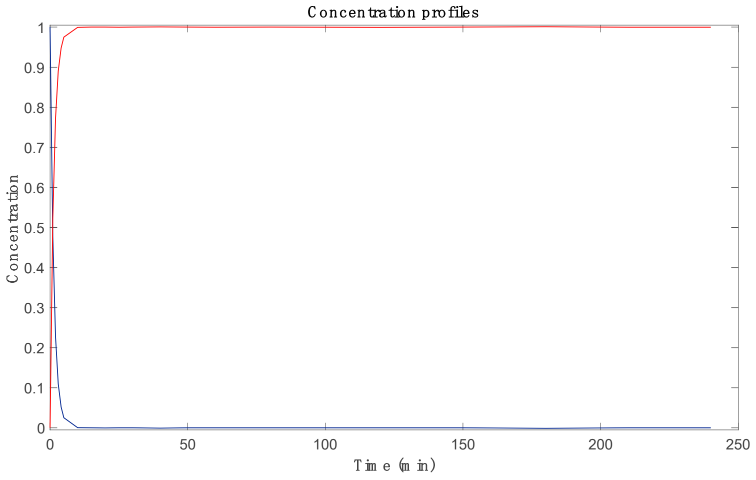

C

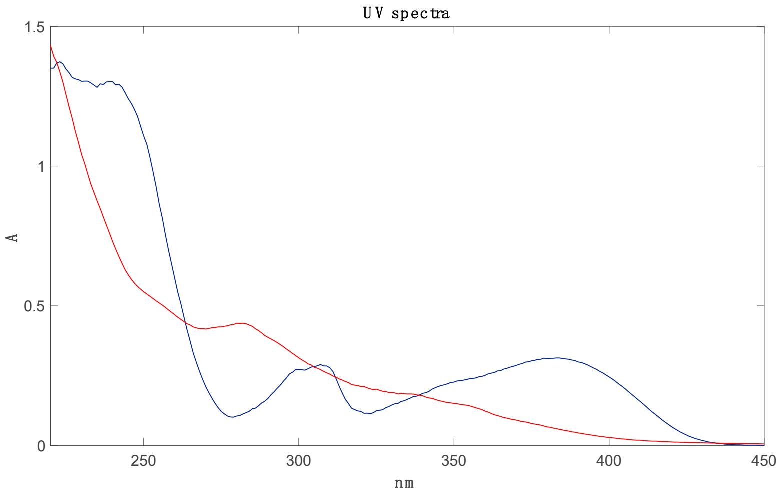

Figure S6. DA6.

A

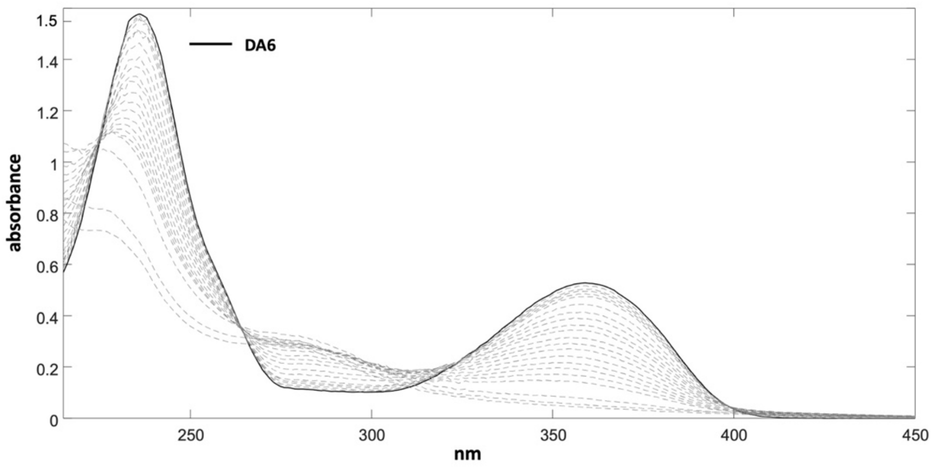

B

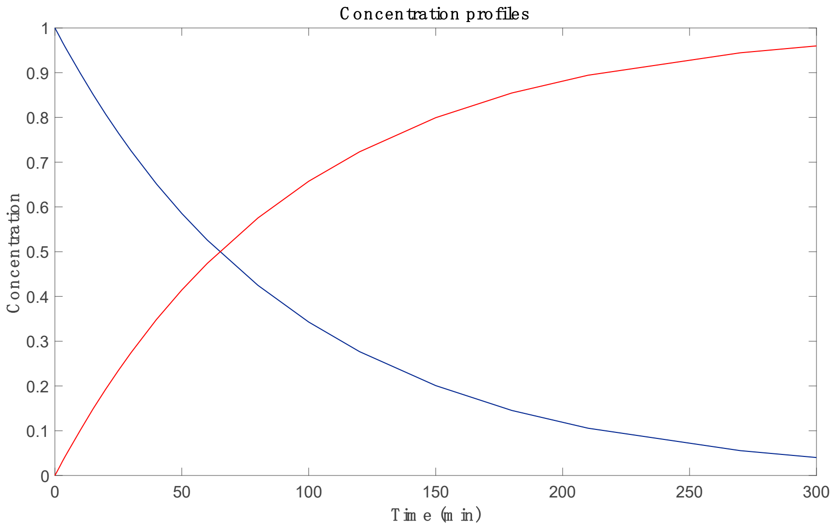

C

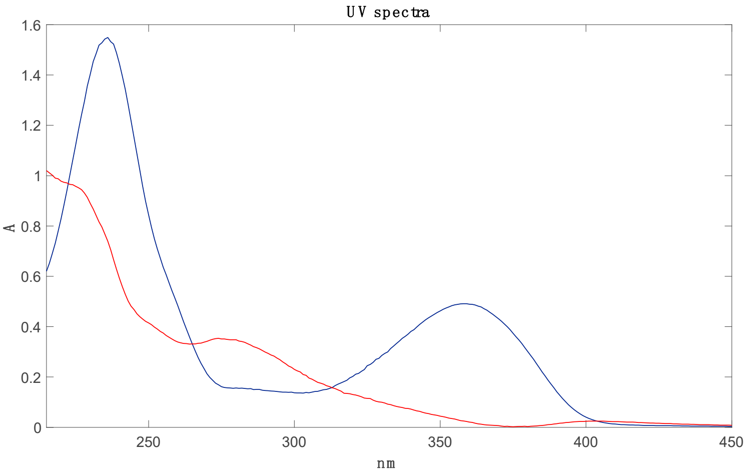

Figure S7. DA7.

A

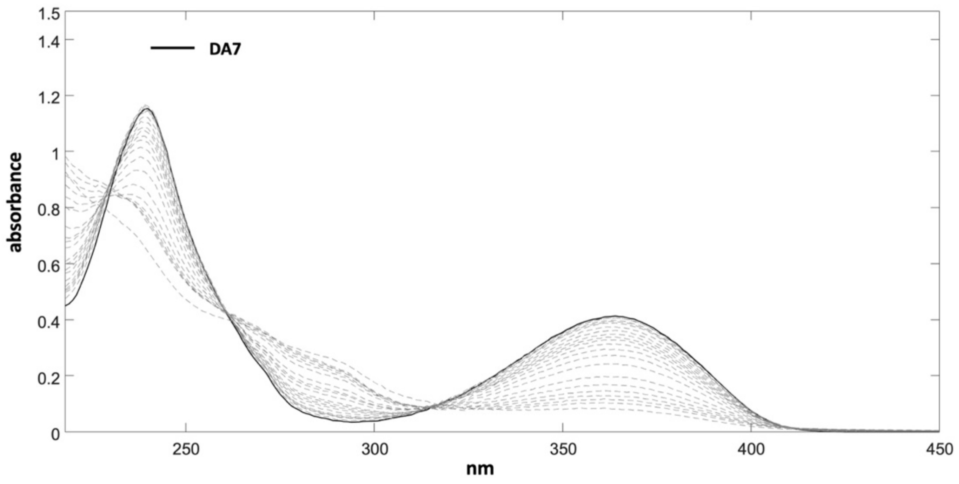

B

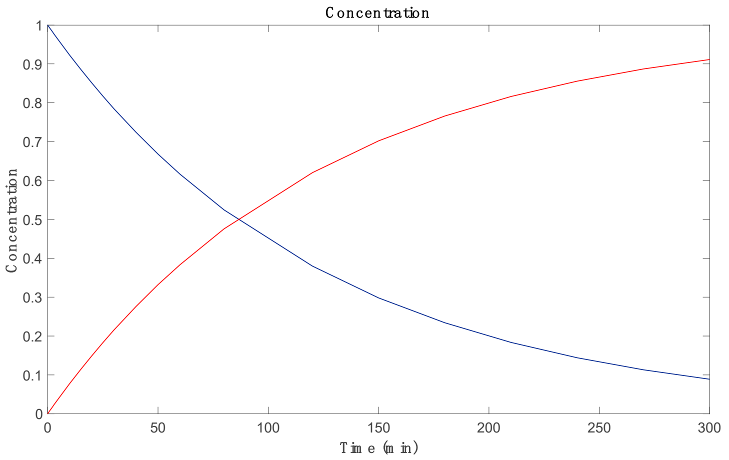

C

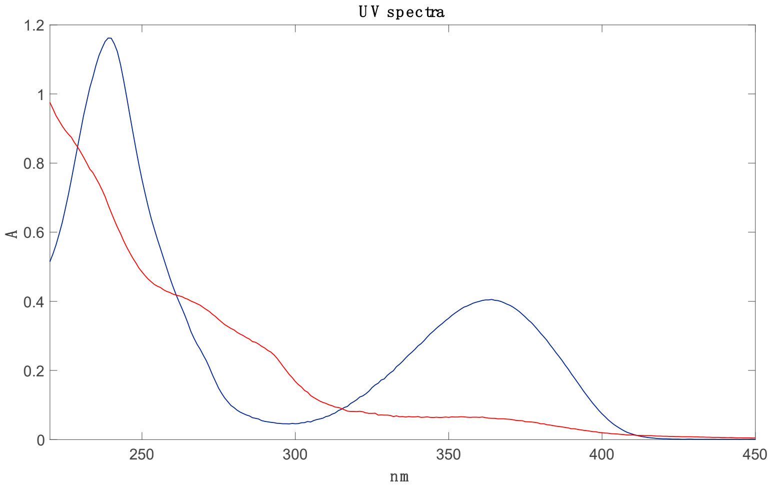

Figure S8. DA8.

A

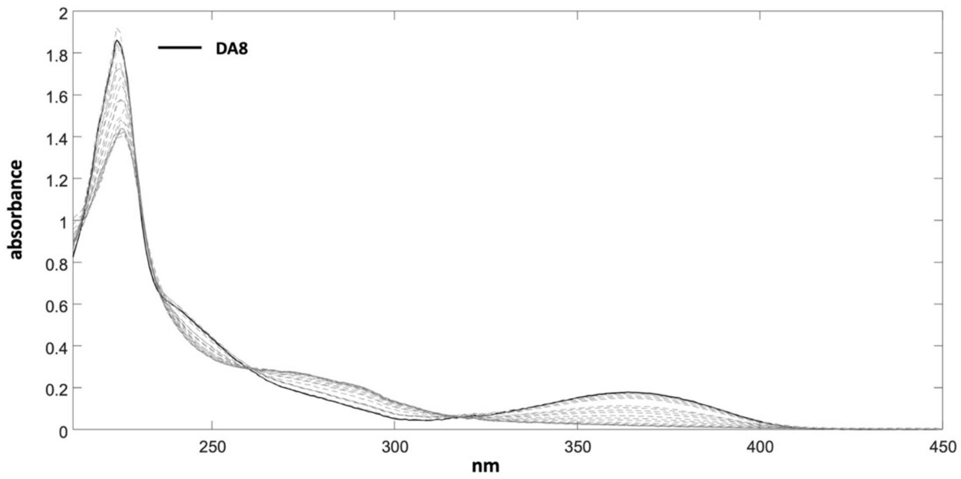

B

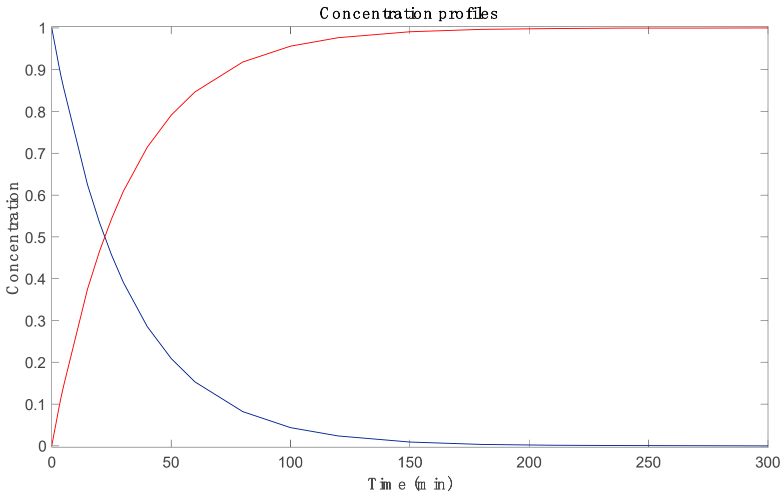

C

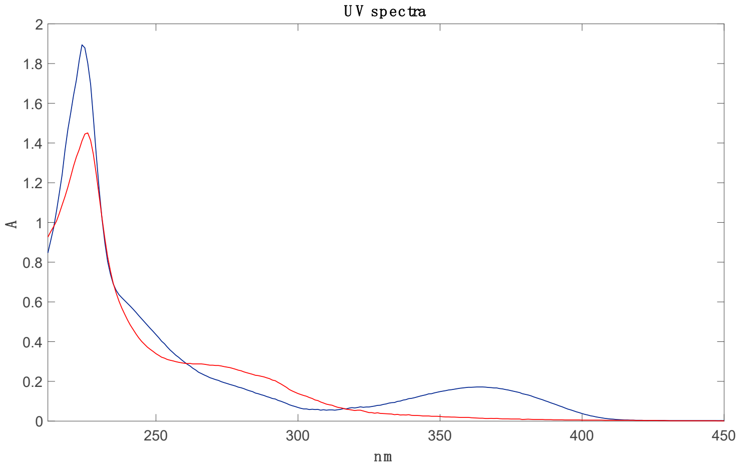

Figure S9. DA10.

A

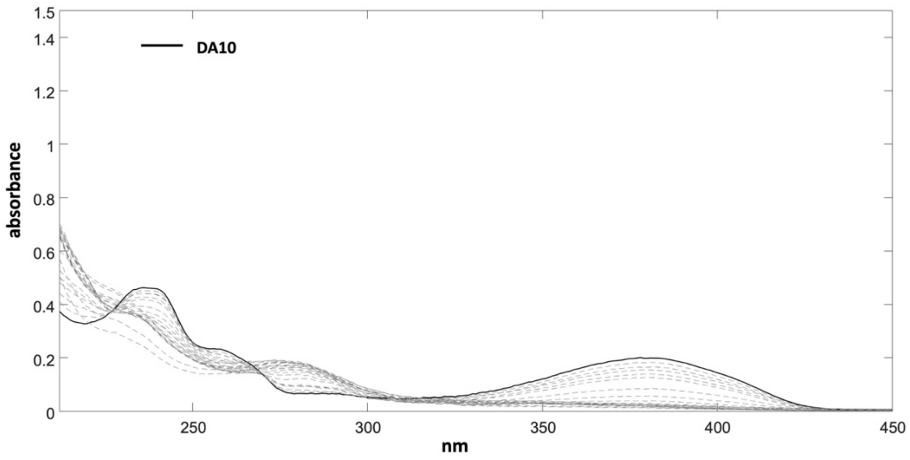

B

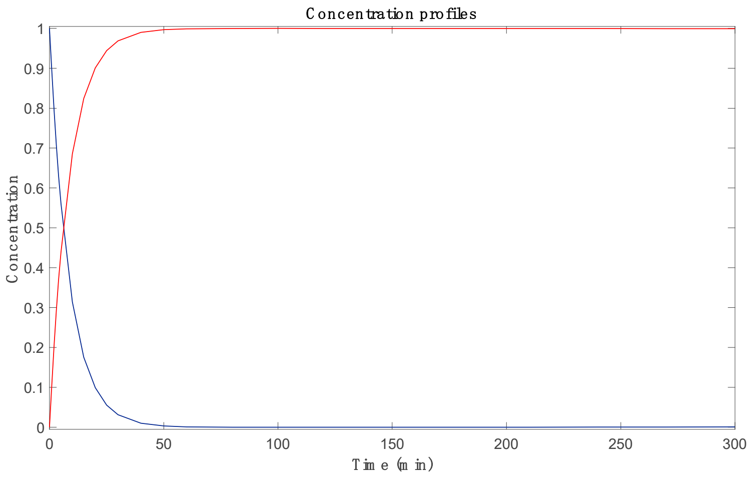

C

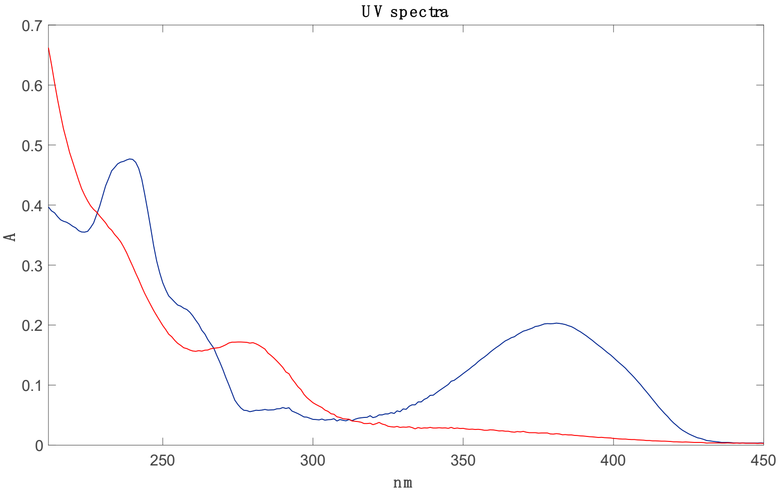

Figure S10. DA11.

A

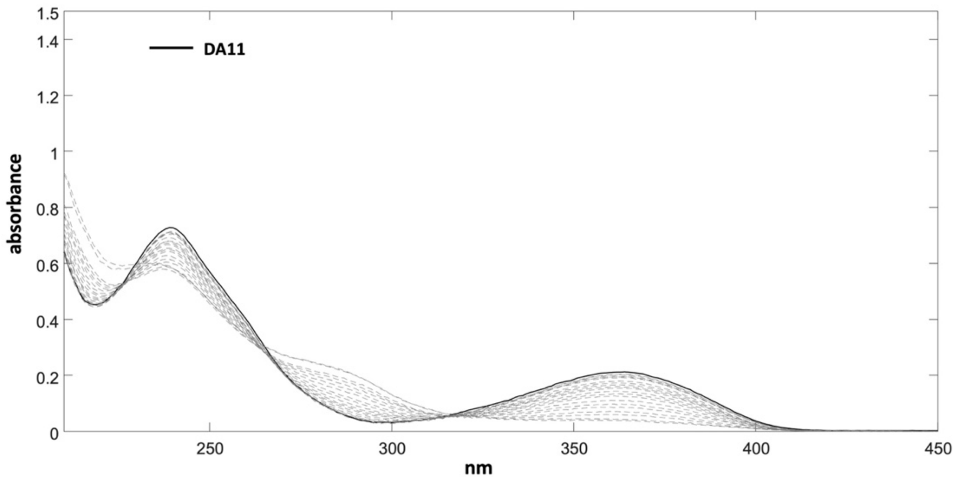

B

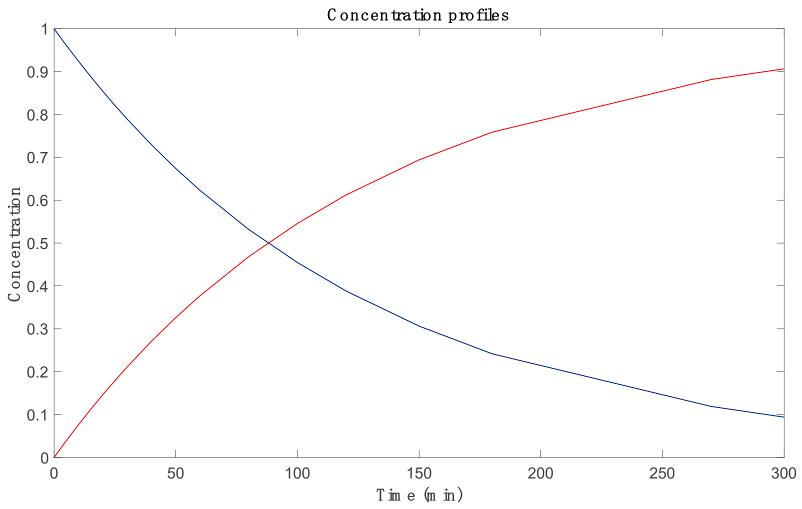

C

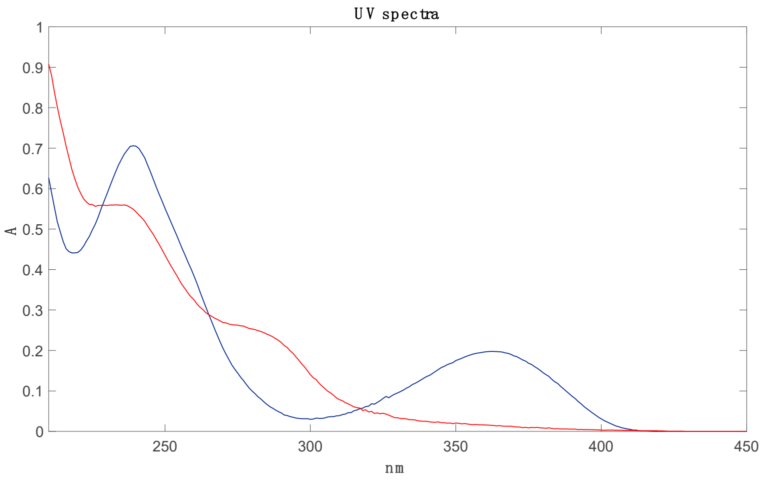

Figure S11. DA12.

A

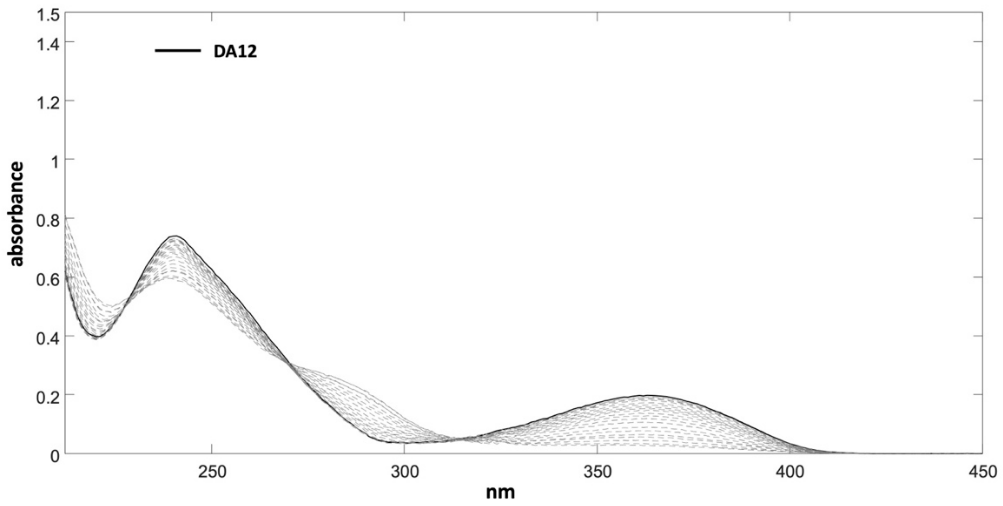

B

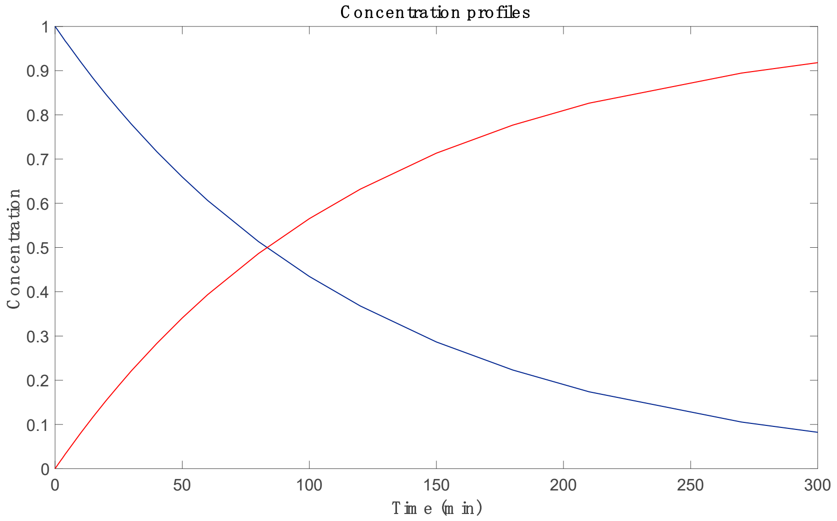

C

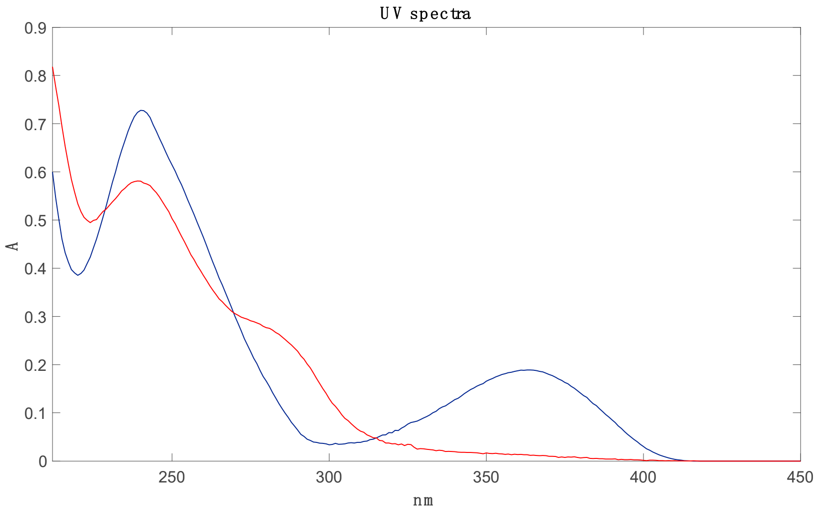

Figure S12. HM8.

A

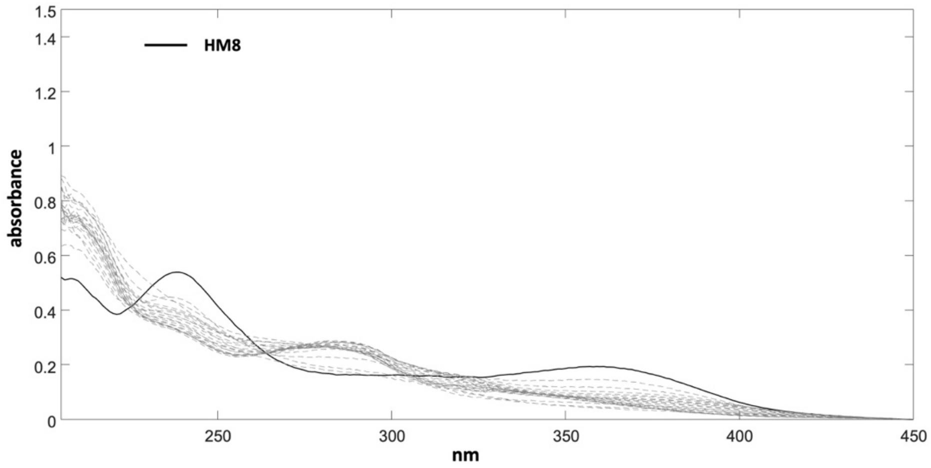

B

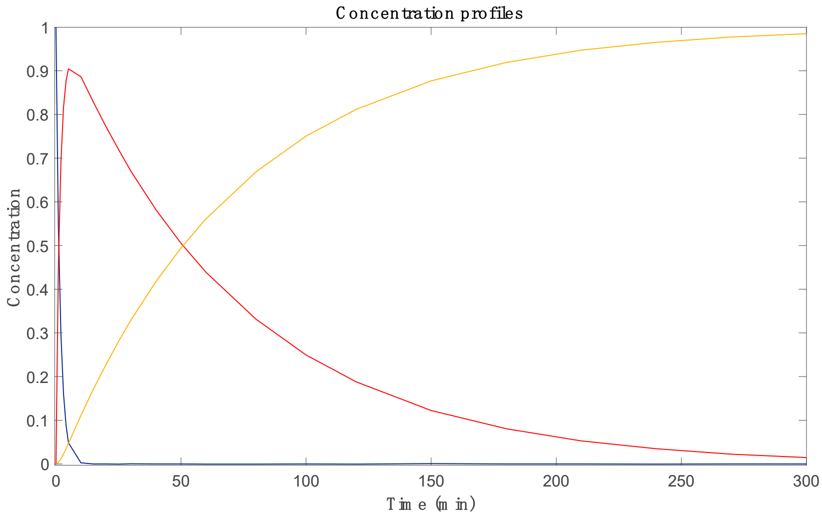

C

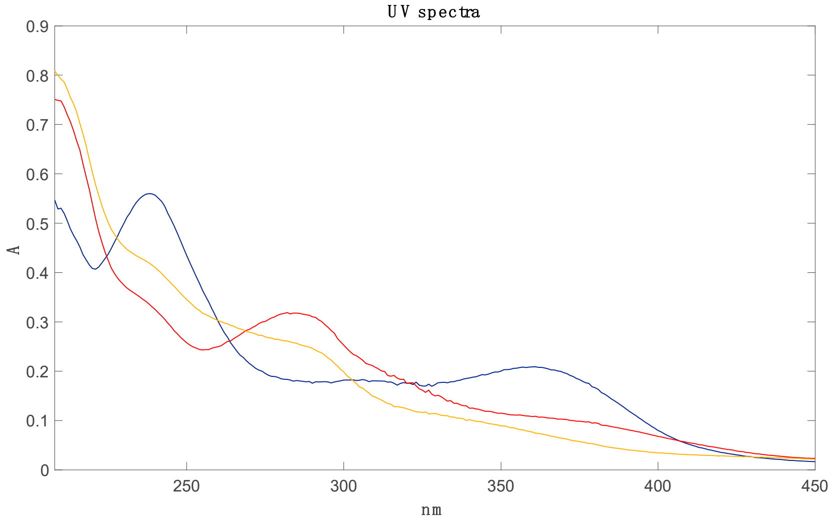

Figure S13. HM10.

A

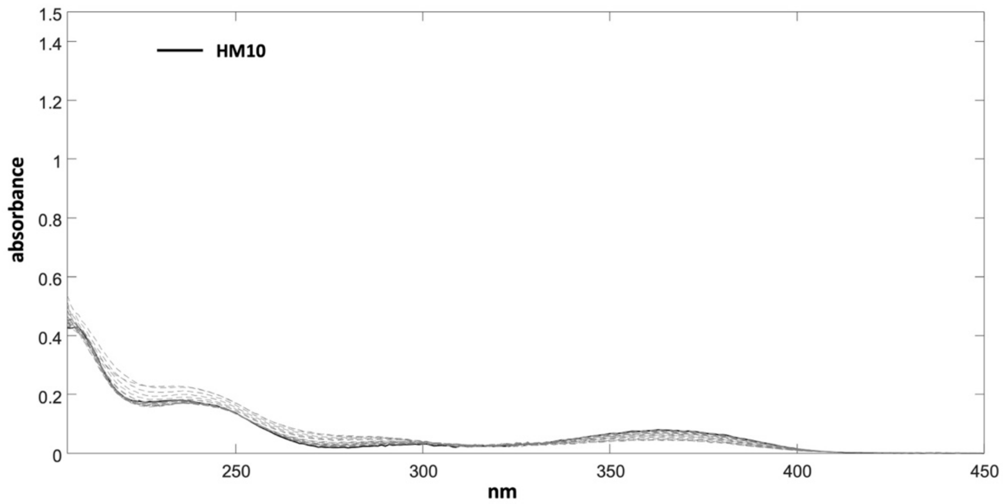

B

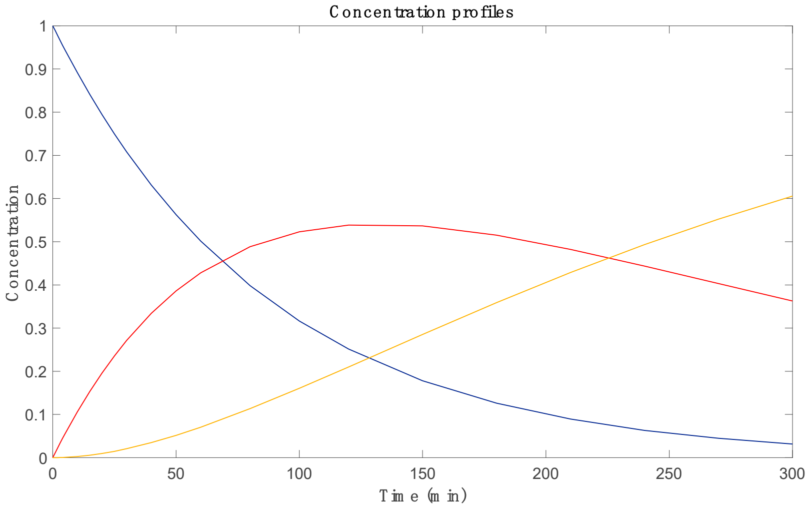

C

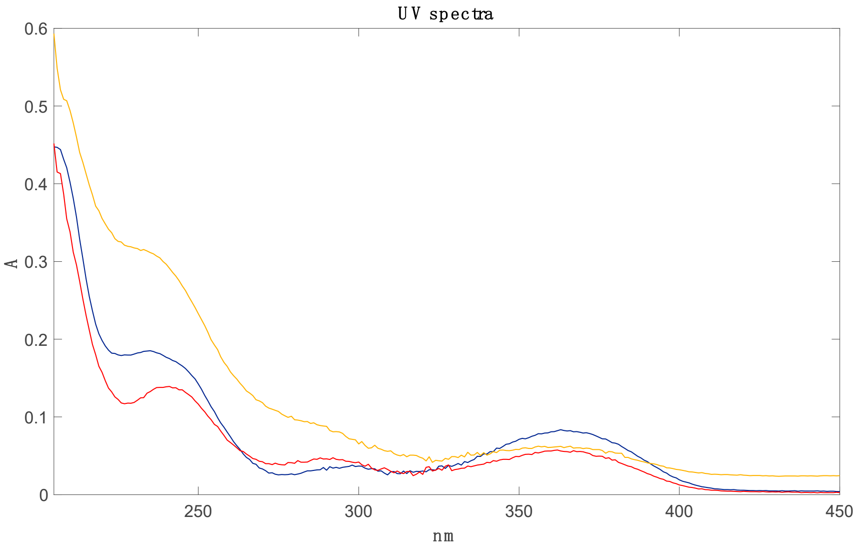

Figure S14. HM13.

A

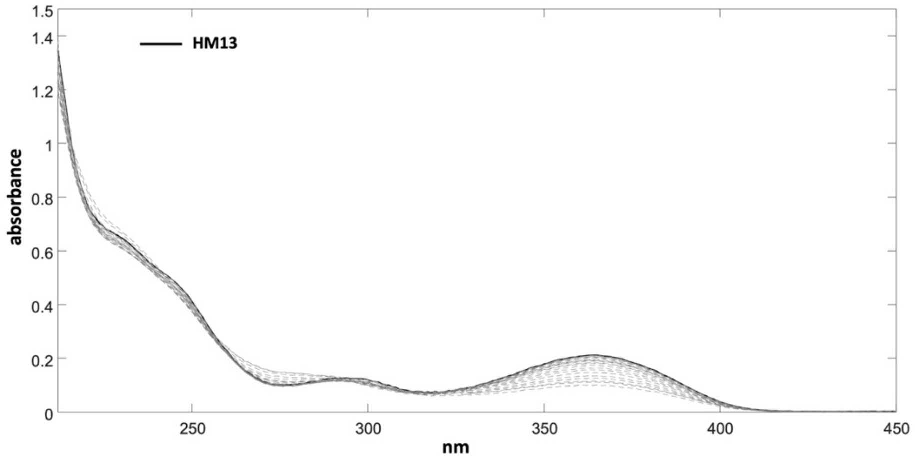

B

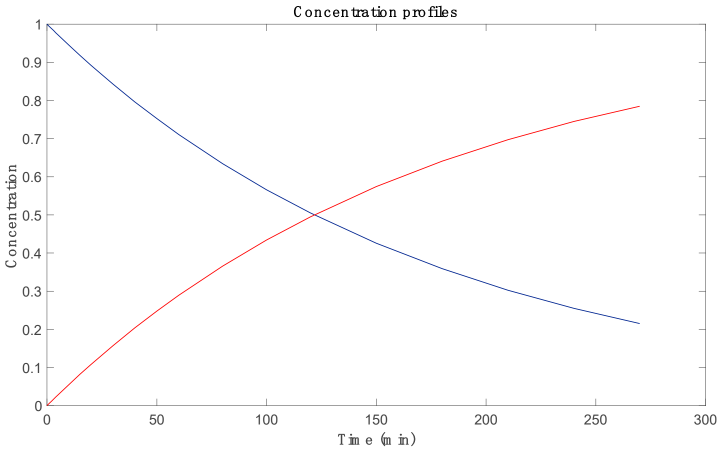

C

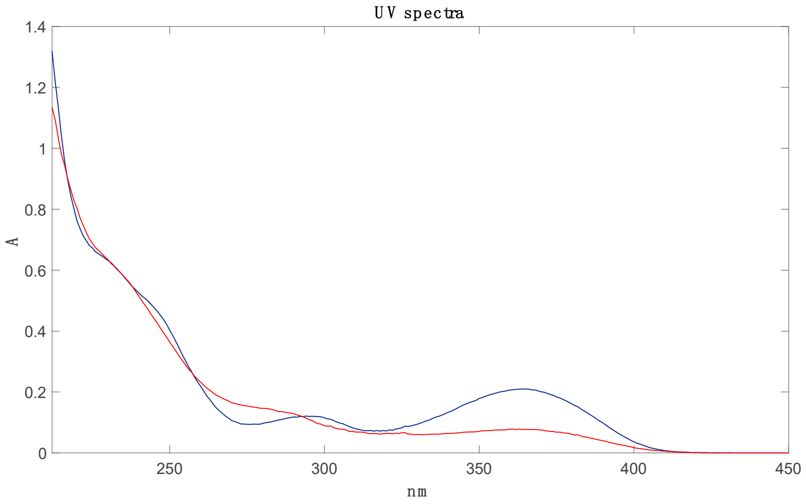

Figure S15. HM14.

A

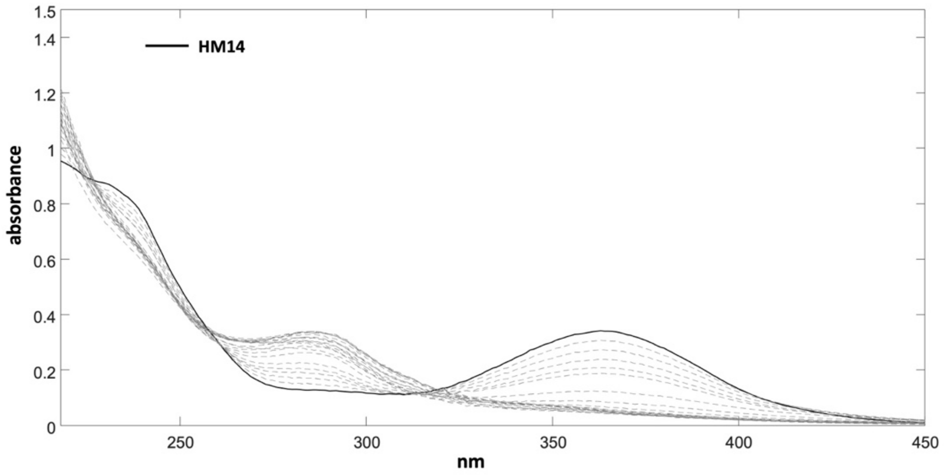

B

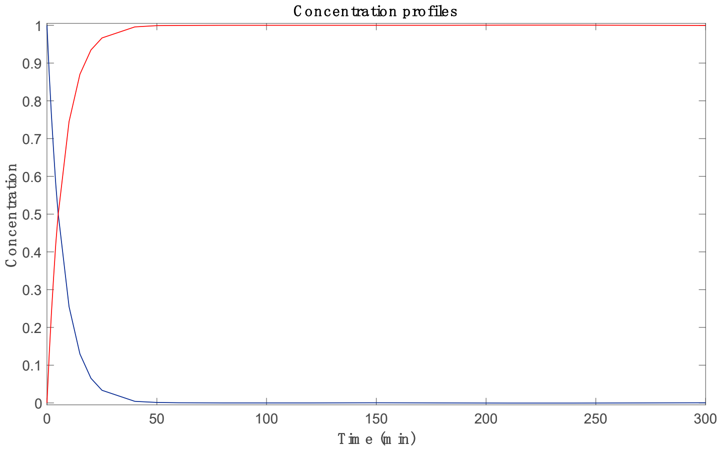

C

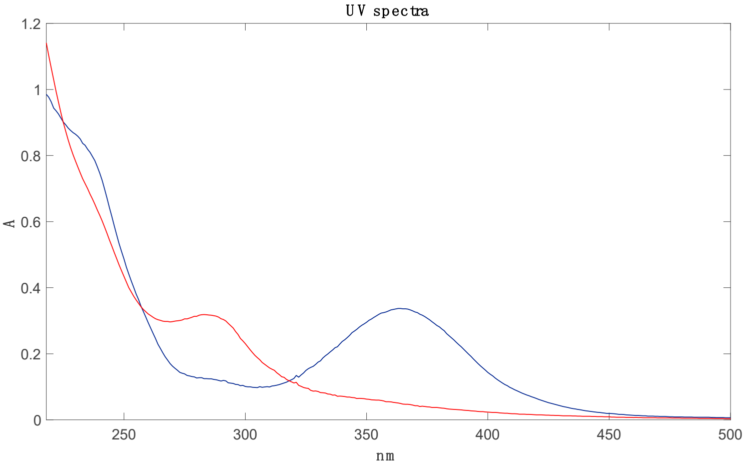

Figure S16. HM15.

A

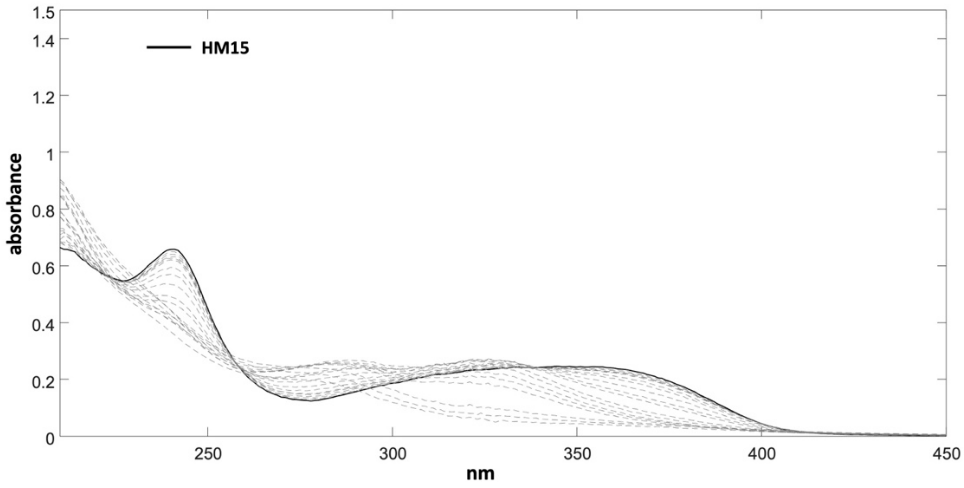

B

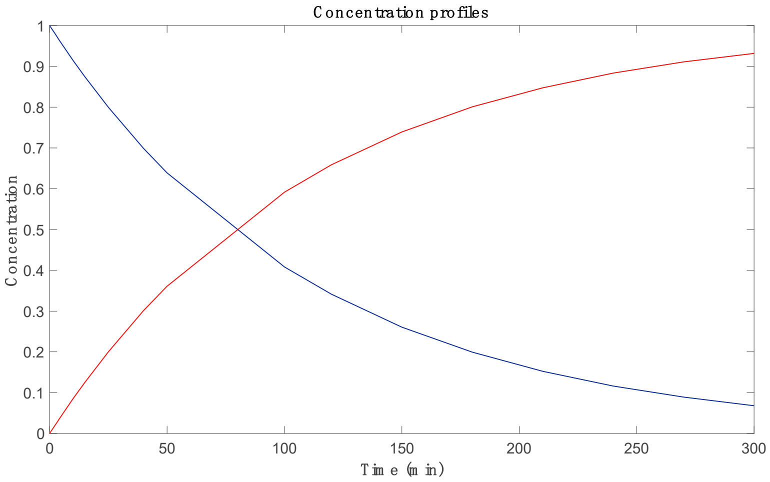

C

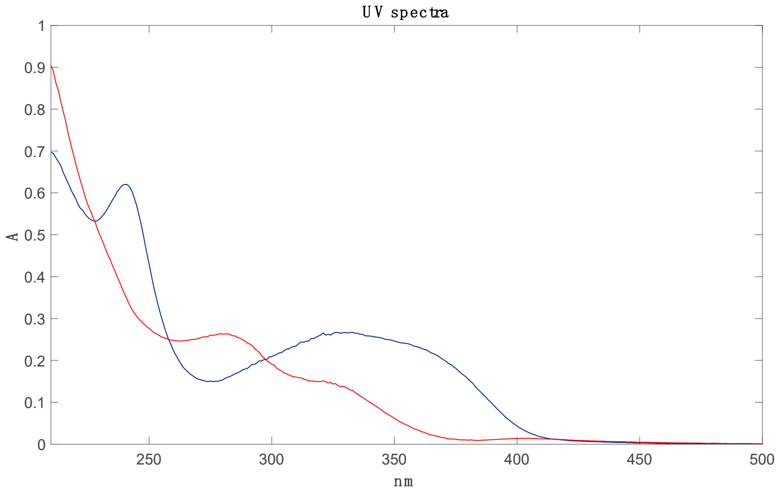

Figure S17. MD20.

A

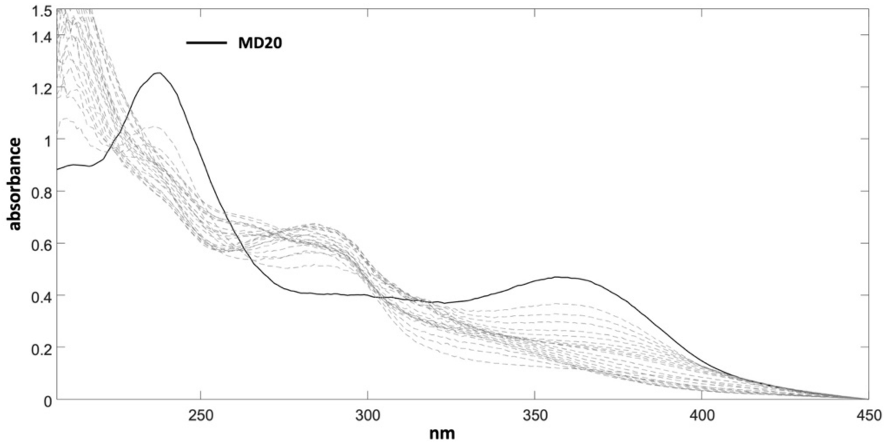

B

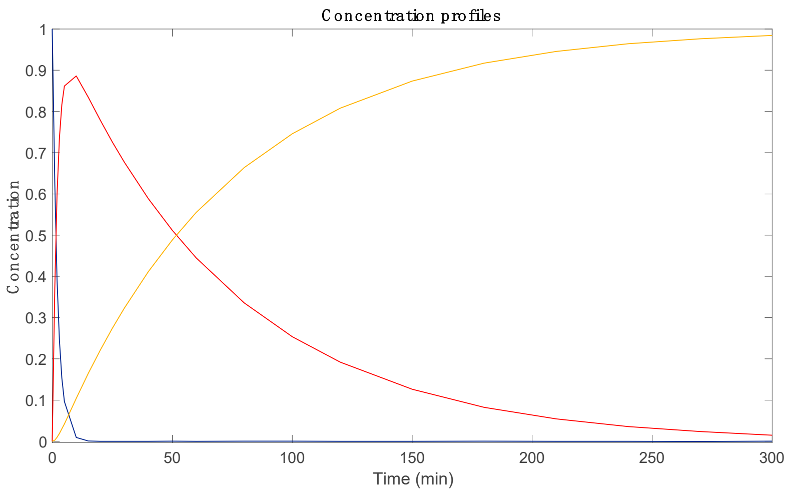

C

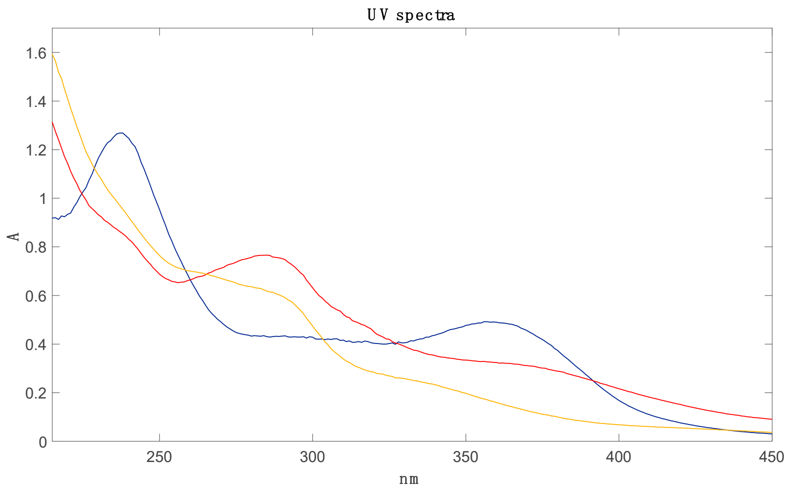

Figure S18. M3.

A

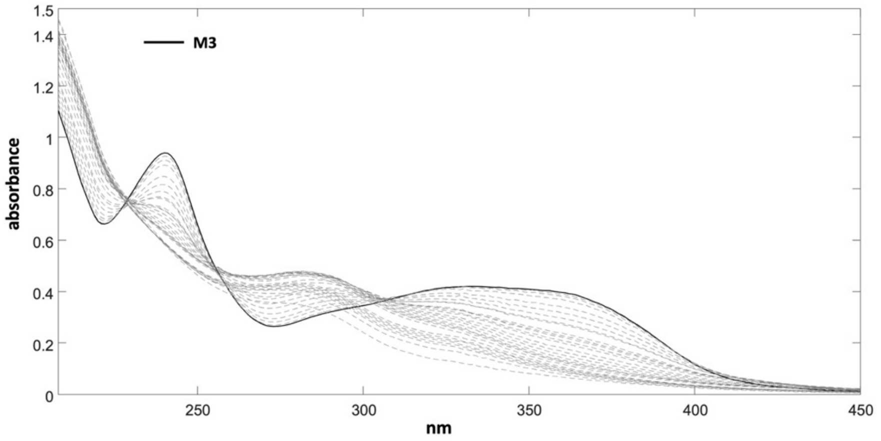

B

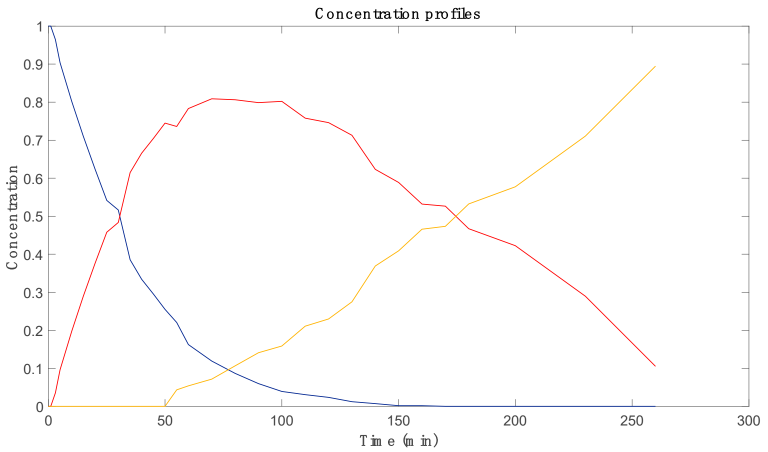

C

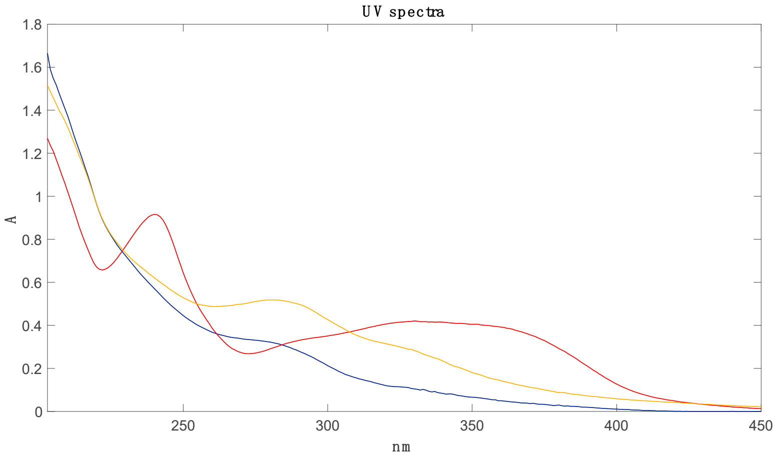

Figure S19. NIMO.

A

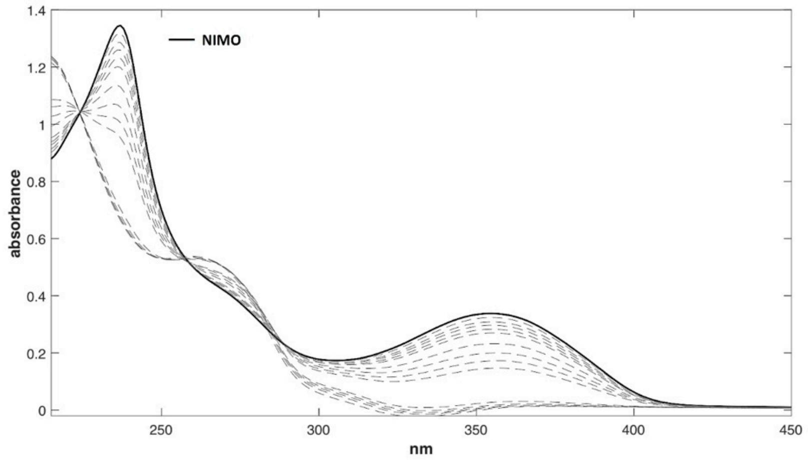

B

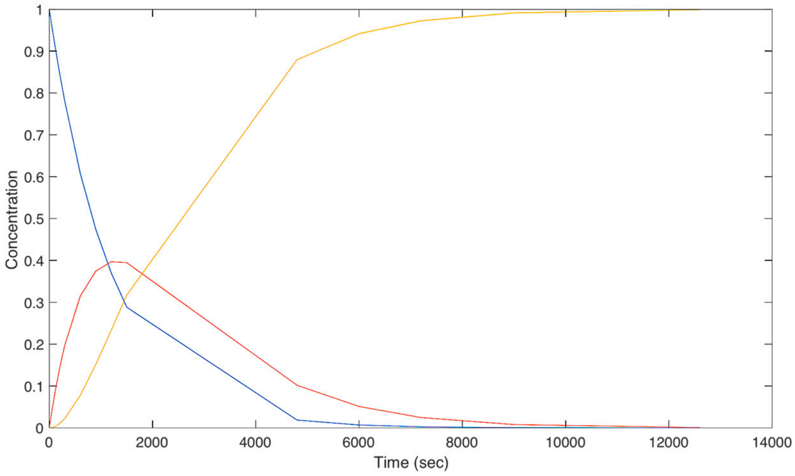

C

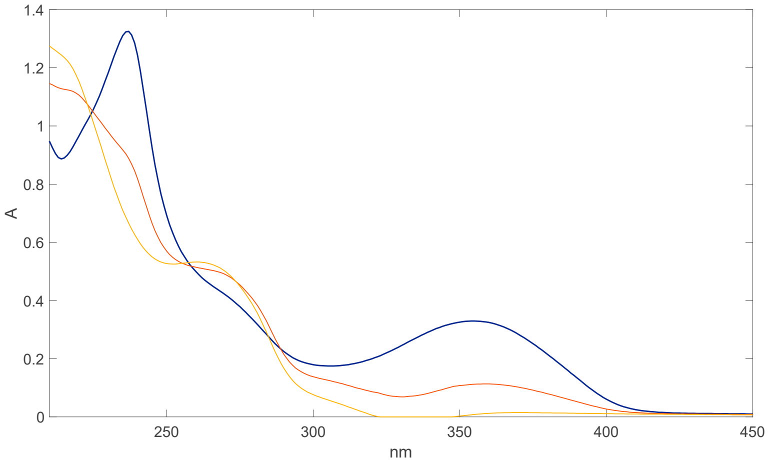

Figure S20. NICA.

A

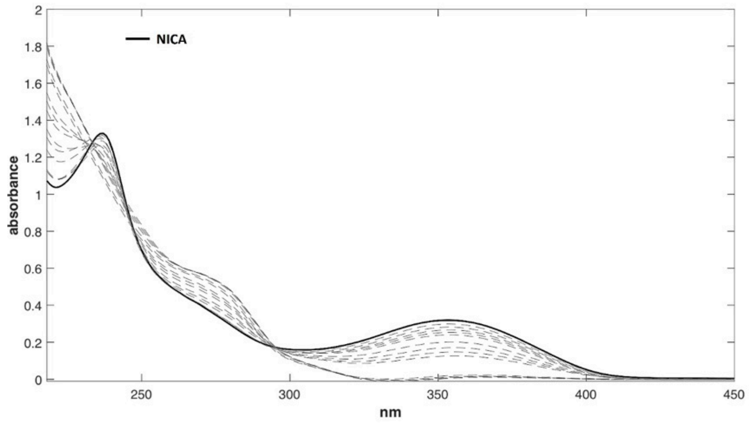

B

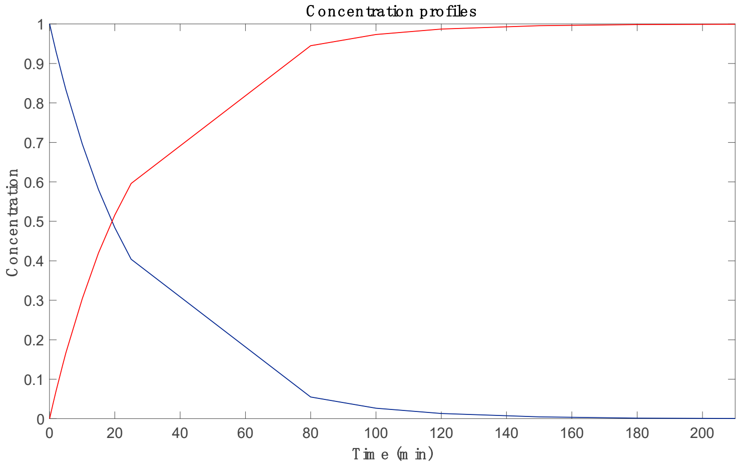

C

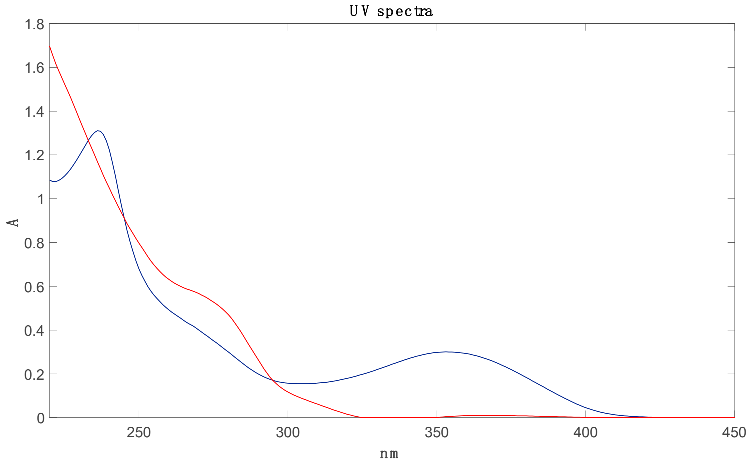

Figure S21. Scores plot.

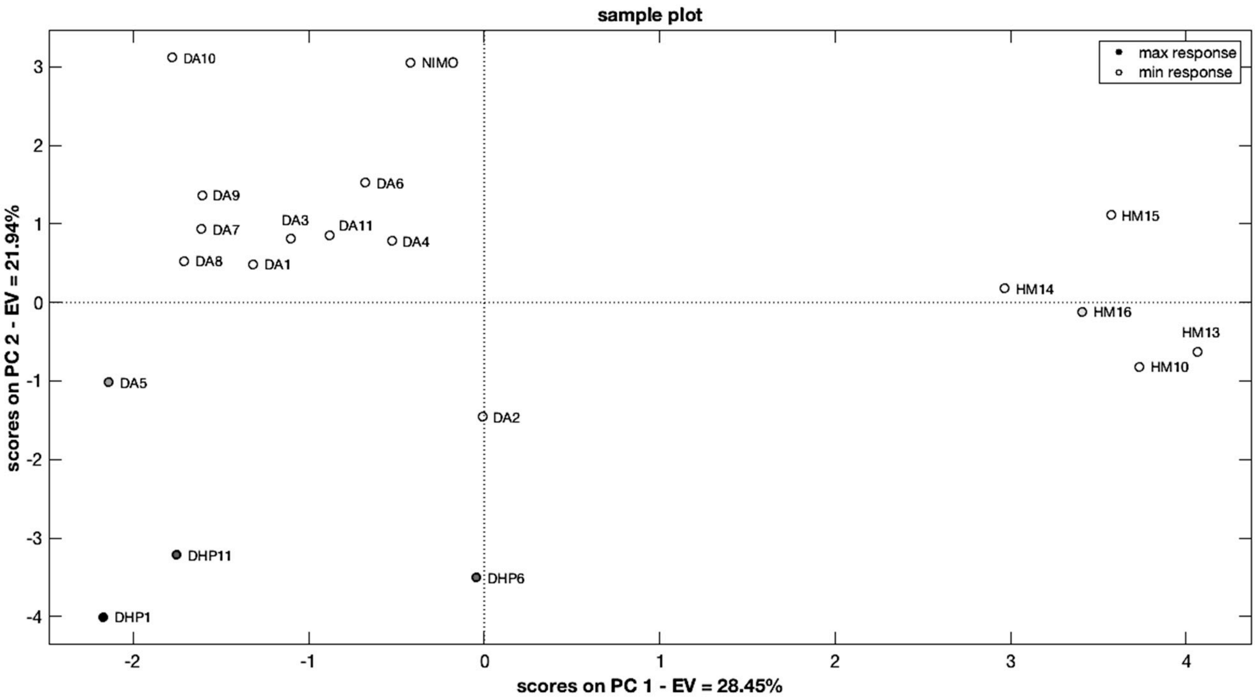

Figure S22. X-loadings plot.

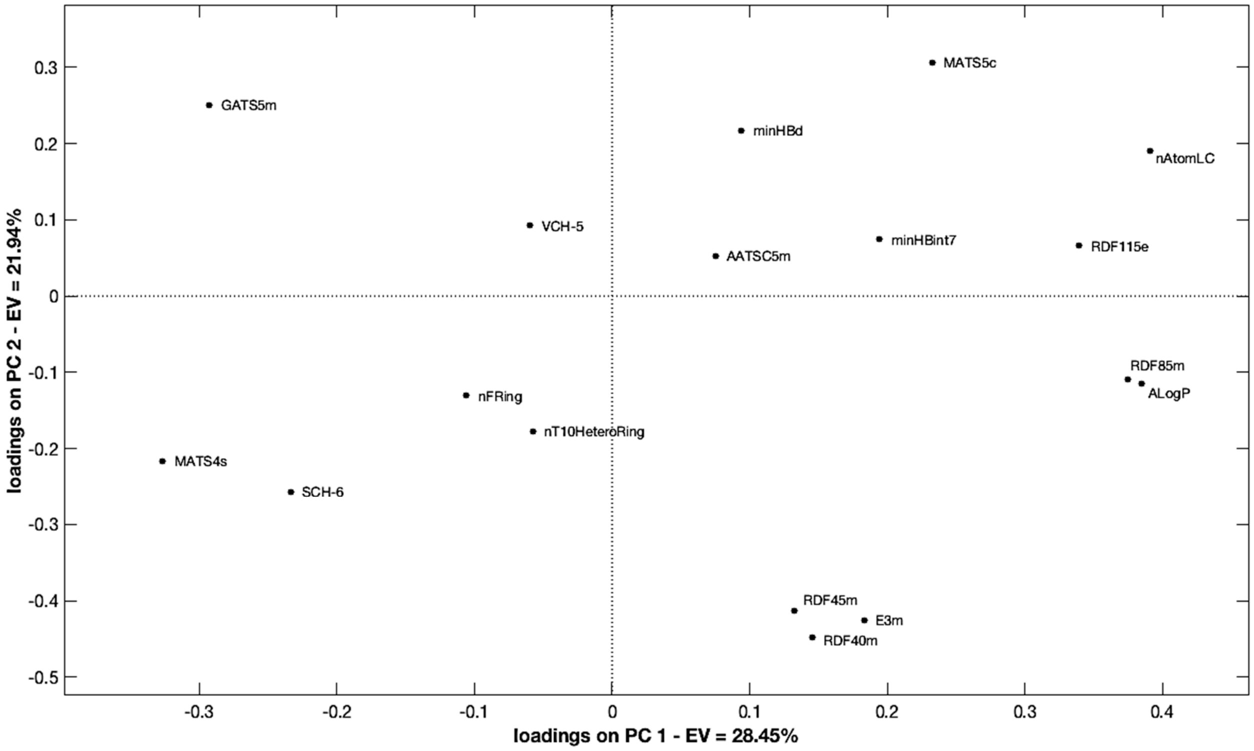

Supplement: Supplementary file 1 [file pharmaceutics-16-00206-s001.zip › pharmaceutics-2814436-supplementary.pdf]
